# ﻿Description of two new species of *Chiloglanis* (Teleostei, Mochokidae) from the Eastern Zimbabwe Highlands freshwater ecoregion: an overlooked hotspot of rheophilic fishes

**DOI:** 10.3897/zookeys.1241.138917

**Published:** 2025-06-16

**Authors:** Tadiwa I. Mutizwa, Taurai Bere, Wilbert T. Kadye, Pedro H. N. Bragança, Albert Chakona

**Affiliations:** 1 NRF-South African Institute for Aquatic Biodiversity (NRF-SAIAB), P. Bag 1015, Makhanda (Grahamstown) 6140, South Africa NRF-South African Institute for Aquatic Biodiversity Makhanda South Africa; 2 Department of Ichthyology and Fisheries Science, Rhodes University, PO Box 94, Makhanda (Grahamstown), 6140, South Africa Rhodes University Makhanda South Africa; 3 School of Wildlife, Ecology and Conservation, Chinhoyi University of Technology, Private Bag 7724, Chinhoyi, Zimbabwe Chinhoyi University of Technology Chinhoyi Zimbabwe; 4 Department of Ichthyology, American Museum of Natural History, Central Park West at 79th Street, New York, NY 10024, USA American Museum of Natural History New York United States of America

**Keywords:** Afromontane streams, diversity, ecological impacts, integrative taxonomy, southern Africa, suckermouth catfishes

## Abstract

Growing evidence indicates that species diversity within the genus *Chiloglanis* Peters 1868 is poorly resolved and major taxonomic revisions are required. By integrating genetic and morphological analyses, this study describes two new *Chiloglanis* species from the Eastern Zimbabwe Highlands (EZH) freshwater ecoregion, a region that, until only recently, had been poorly-explored in terms of its ichthyological diversity. *Chiloglanisasperocutis* Mutizwa, Bragança & Chakona, **sp. nov.** is distinguished from other southern African congeners by a combination of characters, including ridge-like tubercles distributed on the dorsal and lateral surfaces of the head and body giving the skin a conspicuously rough texture, ten closely packed mandibular teeth, deeply forked caudal fin and a high number of primary premaxillary teeth (68–128). *Chiloglaniscompactus* Mutizwa, Bragança & Chakona, **sp. nov.**, attains the smallest size (> 46 mm SL) of all currently known congeners in southern Africa. It is distinguished from all the other congeners from the region by a combination of characters; the possession of seven pectoral fin rays, conical tubercles distributed across the dorsal and lateral surface of the head and body, eight widely spaced mandibular teeth, a shallow forked caudal fin with rounded lobes, a low number of primary premaxillary teeth (31–53) and fewer dorsal fin rays (5). These two species are distributed in both the Buzi and Pungwe River systems. The study is the first in a series of publications that will provide formal descriptions of a number of deeply divergent lineages (candidate species) identified in previous studies from southern Africa. The persistence of the unique riverine fauna in the EZH is threatened by multiple impacts that are altering the hydrological regime of the rivers and streams as well as habitat degradation and excessive sedimentation from gold panning and agricultural activities.

## ﻿Introduction

Previous and ongoing studies indicate that many of the currently recognised freshwater fish species in southern African harbour hidden diversity (e.g., Swartz et al. 2009; Chakona and Swartz 2013; Chakona et al. 2015, 2018; Kambikambi et al. 2021; Mazungula and Chakona 2021; Sithole et al. 2023; Mutizwa et al. 2024; Scheepers et al. 2024). Recently, Chakona et al. (2018) provided the first evidence of undocumented diversity and taxonomic conflicts in fishes from high attitude streams in the Eastern Zimbabwe Highlands freshwater ecoregion (**EZH**), one of the poorly explored regions in southern Africa. Similar findings were reported from a number of studies on species from other high altitude streams in east and west Africa (e.g., Friel and Vigliotta 2011; Schmidt et al. 2015, 2016, 2017, 2023; Morris et al. 2016; Schmidt and Barrientos 2019; Schedel et al. 2024). This indicates that, despite being historically assumed to have a depauperate fish fauna, these high altitude freshwater ecosystems represent previously overlooked hotspots of diversity and endemism (e.g., Schmidt et al. 2017). Growing evidence shows that many groups of fishes inhabiting high altitude streams in Africa are in need of major taxonomic reassessments (e.g., Friel and Vigliotta 2011; Schmidt et al. 2015, 2016, 2017, 2023; Morris et al. 2016; Schmidt and Barrientos 2019).

The aim of the present study was to investigate the taxonomic status of suckermouth catfishes of the genus *Chiloglanis* Peters, 1868 from the EZH. The first published detailed checklist of freshwater fishes of Zimbabwe by Jubb (1961) contained one species of suckermouth catfish, *Chiloglanisneumanni* Boulenger, 1911, from the EZH. For reasons that remain unclear in the literature, subsequent publications such as Bell-Cross (1976) listed two species, *Chiloglanisemarginatus* Jubb and Le Roux, 1969 and *C.neumanni*, whereas Skelton (2001) recognised three species, *C.emarginatus*, *C.neumanni* and *Chiloglanispretoriae* Van der Horst, 1931 from the same region. Marshall (2011), who summarised the existing knowledge of the fishes of Zimbabwe, concluded that the Limpopo River system represented the northern-most distribution limit for *C.pretoriae*, and questioned the taxonomic assignment of the EZH suckermouth catfishes to *C.neumanni*, a species that was described from the Bubu River, a tributary of the Great Ruaha River in Tanzania. Marshall (2011) further commented that *C.neumanni* and *C.emarginatus* are unlikely to occur in Zimbabwe and suggested that specimens that were previously assigned to these two species likely represented several undescribed species whose taxonomic identity required further investigation.

Marshall’s (2011) tentative proposition was supported by subsequent results from DNA barcoding studies that showed considerable genetic divergence of suckermouth catfishes of the EZH from currently described species of *Chiloglanis* from southern Africa (Chakona Et Al. 2018; Mutizwa *et al*. 2024) and specimens from the Great Ruaha River system in Tanzania (Day Et Al. 2023). Chakona et al. (2018) identified at least three candidate species of *Chiloglanis* from the Pungwe and Buzi River systems in the EZH. Here, we apply an integrative taxonomic approach combining genetic and morphological analyses to provide evidence supporting the recognition of two of these lineages tentatively named *Chiloglanis* sp. “rough skin” and *Chiloglanis* sp. “dwarf” in Chakona et al. (2018), as distinct species.

## ﻿Materials and methods

### ﻿Sample collection

**Figure 1.**
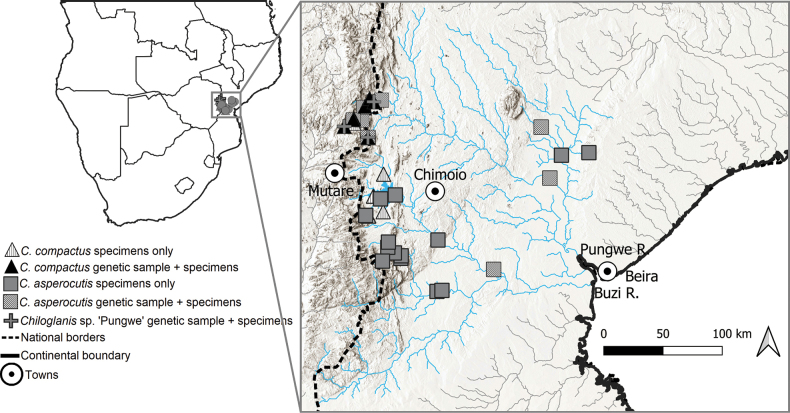
Collection sites of *Chiloglanis* specimens from the Pungwe and Buzi river systems.

Voucher specimens and tissue samples were collected during surveys of the EZH conducted in 2013, 2014 and 2022 in the Buzi and Pungwe river systems (Fig. 1). Sampling was done using a Samus-725M electrofisher with a seine net placed downstream to capture immobilised fish in fast-flowing current. Species were identified using regional identification guides by Skelton (2001) and Marshall (2011). Captured fishes were euthanized with clove oil, some specimens were digitally photographed, and a small piece of muscle tissue was dissected from the right side of each specimen and preserved in 95% ethanol. Tissue samples were stored at -80 °C at the National Research Foundation-South African Institute for Aquatic Biodiversity (**NRF-SAIAB**), Makhanda. Voucher specimens were fixed in 10% formalin in the field. They were then transferred through 10% and 50% to 70% ethanol for long-term storage. All voucher specimens were deposited into the fish collection facility at the NRF-SAIAB as reference material. In addition, the study included specimens collected from the Pungwe and Buzi Rivers prior to the 2013 that were lodged at the National Fish Collection at NRF-SAIAB (see Material examined).

### ﻿DNA extraction, amplification, and sequencing

This study used sequences generated by Chakona et al. (2018) and Mutizwa et al. (2024) deposited in GenBank (accession numbers: MH432018–MH432062, PP156890–PP156895). An additional eight COI sequences were generated for this study using specimens collected from tributaries of the Pungwe River in Gorongosa National Park, Mozambique in surveys conducted between July and August 2022 (GenBank accession numbers: PQ424602–PQ424609). Preparation and sequencing of genetic material was done in the Aquatic Genomics Research Platform at the NRF-SAIAB. DNA extraction, amplification, and sequencing followed Mutizwa et al. (2024). Genomic DNA was extracted from preserved tissues using the salting-out method (Sunnucks and Hales 1996). The mitochondrial DNA cytochrome c oxidase subunit I (COI) gene was amplified by polymerase chain reaction (PCR) using the universal fish DNA barcoding primer set FishF1 and FishR1 (Ward et al. 2005). PCRs were performed with a Veriti 96 well thermal cycler (Applied Biosystems, USA) and each reaction mixture (25 µL) contained 50–100 ng) of template DNA, 6.5 µL of water, 0.5 µL of each primer (10 µM), and 12.5 µL Taq DNA polymerase 2 × master mix red (Amplicon PCR enzymes and reagents, Denmark). The PCR amplification profile had an initial denaturation step of 3 min at 94 °C followed by 38 cycles of 30 sec at 94 °C, annealing at 55 °C for 30 sec, and extension at 72 °C for 50 sec, and final extension at 72 °C for 7 min. The amplicons were purified using an Exonuclease I-Shrimp Alkaline Phosphate (Exo/SAP, Thermo Fisher Scientific, USA) protocol (Werle et al. 1994), sequenced using standard fluorescent BigDye v. 3.1 (Applied Biosystems, USA) terminator chemistry in the forward direction, and analysed on a 3500 Genetic Analyser (Applied Biosystems, USA) at the NRF-SAIAB. Locality details for *Chiloglanis* sp. “dwarf”, *Chiloglanis* sp. “rough skin” and comparative sequences from southern African *Chiloglanis* species and outgroup species are given in the Suppl. material 1: table S1).

### ﻿Phylogenetic analyses

Phylogenetic relationships among *Chiloglanis* species from southern Africa were inferred using Bayesian and maximum likelihood approaches. Mitochondrial DNA sequences were edited, aligned, and trimmed in MEGA-X (Kumar et al. 2018). The sequences were translated into amino acid sequences in MEGA-X to check for stop codons and gaps to ensure that they were copies of functional mitochondrial protein coding sequences. Haplotype groups were identified using DNASP 6 (Rozas et al. 2017). Bayesian analysis was performed using MrBayes 3.1.2 (Ronquist et al. 2012). The dataset was partitioned by codon position and sampled using a reversible-jump Markov chain Monte Carlo (RJ-MCMC), with time-reversible substitution models and gamma distributed rate heterogeneity (Huelsenbeck et al. 2004). Two parallel analyses of four Markov chains were ran for 10 million generations, sampling of trees was done every 1000 generations discarding the first 25% of trees as burn-in. An average standard deviation of split frequencies of < 0.05 indicated the convergence of the two runs in MrBayes. Effective Sample Size (ESS) values and the potential-scale reduction factor for all parameters examined were > 100 and 1.0, respectively. Resulting trees were visualized in FigTree, v. 1.4.3 (Rambaut 2016). Maximum likelihood (ML) analysis of the same dataset was performed in IQ-TREE (Nguyen et al. 2015) through the webserver at http://iqtree.cibiv.univie.ac.at/ (Trifinopoulos et al. 2016). A total of 1000 Ultrafast bootstraps were performed (Minh et al. 2013). Bootstrap values equal to or higher than 70% (Hillis and Bull 1993), and posterior probability values at 0.95 or higher (Alfaro and Holder 2006) were considered to indicate well supported nodes.

### ﻿Morphological analysis

**Figure 2.**
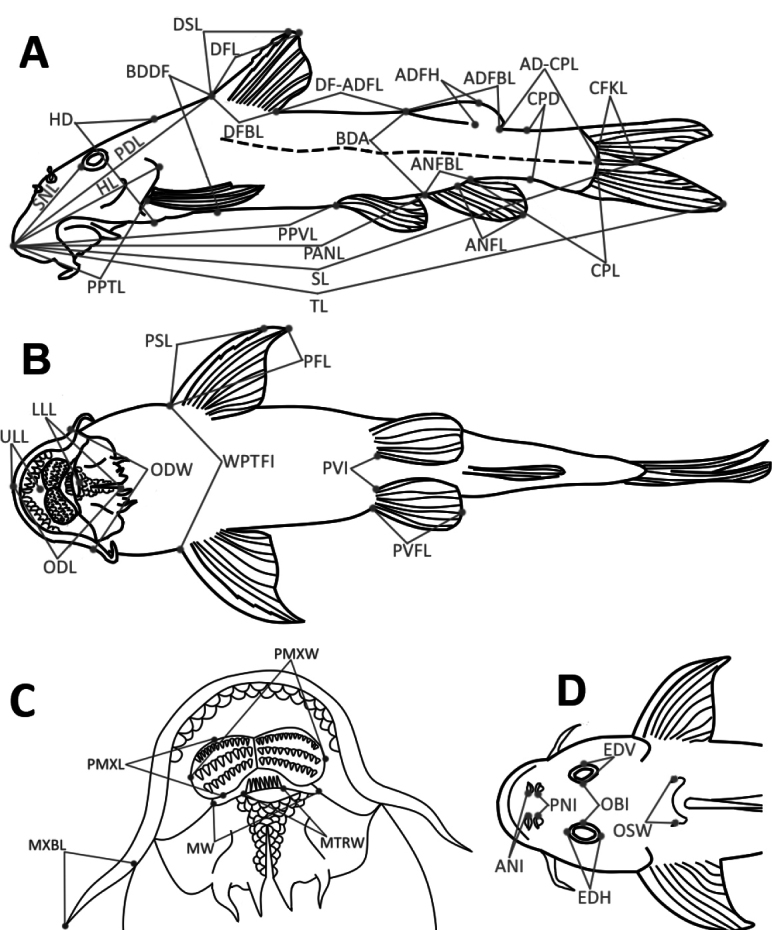
Illustrations of linear measurements recorded from *Chiloglanis* specimens **A** lateral view **B** ventral view of the body **C** ventral view of the oral disc **D** dorsal view of the head. Character abbreviations are explained in the morphological analysis section.

The study examined 110 specimens of *Chiloglanis* sp. “rough skin”, 55 specimens of *Chiloglanis* sp. “dwarf”, and five specimens of *Chiloglanis* sp. “Pungwe” from the EZH. These specimens were compared to their southern African congeners using raw data from Mutizwa et al. (2024). A total of ten counts and 43 measurements (Fig. 2) were considered for this study following Friel and Vigliotta (2008) and Skelton and White (1990). The morphometric characters are defined in Suppl. material 1: table S2). Measurements were taken to the nearest 0.1 mm using digital callipers. Postcranial meristics (i.e., total vertebrae, abdominal vertebrae, and caudal vertebrae) were taken from radiographs following criteria described by Skelton and White (1990). Vertebrae counts excluded the Weberian vertebrae. The ural centrum was counted as a single element. Abdominal vertebrae included vertebrae anterior to the leading anal-fin pterygiophore. The caudal vertebrae included all vertebrae posterior to the leading anal-fin pterygiophore. The specimens were sexed by external examination. Males possess an elongated and pointed genital papilla, while females are distinguished by possession of longitudinal invagination between the anus and the uro-genital papilla (Skelton 2001).

## ﻿Results

### ﻿Molecular analysis

**Figure 3.**
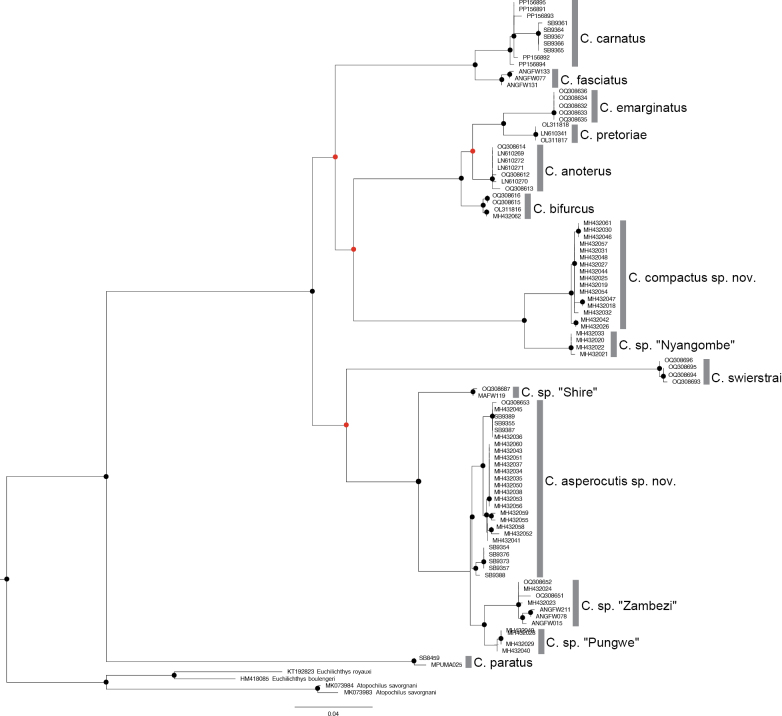
Maximum likelihood tree of the species and lineages of the genus *Chiloglanis* found in southern African based on cytochrome oxidase I (COI). The grey bars represent sequences belonging to the same species. Node colours indicate bootstrap values: black circle ≥ 70; red circle < 70.

**Figure 4.**
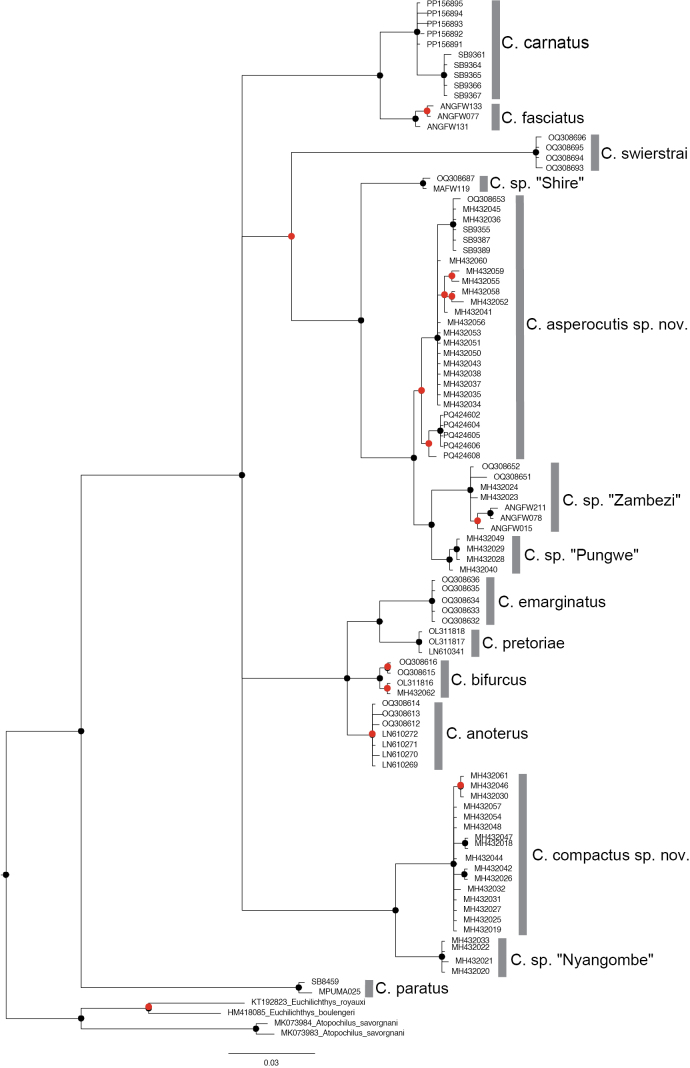
Bayesian inference tree of the species and lineages of the genus *Chiloglanis* found in southern African based on cytochrome oxidase I (COI). The grey bars represent sequences belonging to the same species. Node colours indicate Bayesian posterior probabilities: black circle ≥ 0.95; red circle < 0.95..

The Maximum Likelihood analysis based on 97 sequences (525 base pairs) recovered 14 distinct clades (Fig. 3). These clades corresponded to the eight species from the genus *Chiloglanis* that are currently recognised in southern Africa. The remaining clades correspond to the six candidate species that were identified in previous studies and include the two taxa, *Chiloglanis* sp. ‘rough skin’ and *Chiloglanis* sp. ‘dwarf’ whose taxonomic integrity is the subject of investigation for the present study (Fig. 3). A similar number of clades was recovered from the Bayesian Inference analysis (Fig. 4). The two lineages, *Chiloglanis* sp ‘rough skin’ and *Chiloglanis* sp. ‘dwarf’ were recovered as well-supported monophyletic clades (Figs 3, 4) that are deeply divergent (10.1–18.5%) from all the formally described congeneric species in southern Africa (Table 1). *Chiloglanis* sp. “dwarf” was deeply divergent from *Chiloglanis* sp. “rough skin” (11.8–13.5%, Table 1). Intraspecific genetic divergence was low in *Chiloglanis* sp. “dwarf” (0–0.8%) and *Chiloglanis* sp. “rough skin” (0–1.7%). *Chiloglanis* sp. “dwarf” was recovered as sister to an undescribed lineage from the Zambezi River, *Chiloglanis* sp. “Nyangombe” with a genetic divergence of 4.1–4.8%. *Chiloglanis* sp. “rough skin” sp. nov. was recovered as sister to a clade with undescribed lineages from the Pungwe River, *Chiloglanis* sp. “Pungwe”, and the neighbouring Zambezi River, *Chiloglanis* sp. “Zambezi”. There was relatively low genetic divergence between *Chiloglanis* sp. “rough skin”, *Chiloglanis* sp. “Pungwe” (1.7–3.3%) and *Chiloglanis* sp. “Zambezi” (2.7–4.1%). However, the low genetic divergence between *Chiloglanis* sp. “rough skin”, *Chiloglanis* sp. “Pungwe”, and *Chiloglanis* sp. “Zambezi” was in some cases higher than the interspecific genetic divergence found between some valid southern African *Chiloglanis* species. For example, *C.anoterus* Crass, 1960 and *C.bifurcus* Jubb and Le Roux, 1969 were separated by 2.5–2.9% genetic divergence while *C.emarginatus* and *C.pretoriae* were separated by 3.7% (Table 1). The clade formed by *Chiloglanis* sp. “rough skin”, *Chiloglanis* sp. “Pungwe” and *Chiloglanis* sp. “Zambezi” was separated from *Chiloglanis* sp. “Shire” a lineage from the Shire River by a genetic divergence of 4.7–6.2%. Both *Chiloglanis* sp. “rough skin” and *Chiloglanis* sp. “dwarf” occurred in sympatry within the Buzi River system. Within the Pungwe River, *Chiloglanis* sp. “rough skin” and *Chiloglanis* sp. “dwarf” co-occurred with *Chiloglanis* sp. “Pungwe” (Fig. 1). These results further corroborate the presence of undescribed *Chiloglanis* lineages, such as *Chiloglanis* sp. “Nyangombe” from the Nyangombe River (Zambezi River system), *Chiloglanis* sp. “Shire” from the Shire River (Zambezi River system), *Chiloglanis* sp. “Pungwe” from the Pungwe River and *Chiloglanis* sp. “Zambezi” from the Zambezi River that were identified in previous studies.

**Table 1.** Ranges of cytochrome oxidase I (COI) genetic distances (%) between the *Chiloglanis* species included in the present study.

		1	2	3	4	5	6	7	8	9	10	11	12	13	14	15	16	17
**1**	*C.carnatus*	0–1.7																
**2**	*C.fasciatus*	2.9–3.7	0.2–0.6															
**3**	*C.swierstrai*	14.6–15.5	14.6–15.4	0.0–0.4														
**4**	*C.emarginatus*	11.7–12.2	10.3–10.5	14.9–15.4	0													
**5**	*C.bifurcus*	10.8–11.7	9.9–10.6	14.5–15.5	5	0.0–0.4												
**6**	*C.anoterus*	10.8–11.7	9.9–10.6	14.0–14.7	4.4–5.0	2.5–2.9	0.0–0.6											
**7**	*C.pretoriae*	11.7–12.4	10.5–10.8	14.7–15.2	3.7	4.3	3.5–3.9	0										
**8**	*C.paratus*	16.0–16.7	15.4–16.4	18.1–18.9	17.3–17.8	14.6–15.6	14.9–15.8	15.3–15.6	0.0–0.6									
**9**	***Chiloglanis*compactus sp. nov.**	13.2–14.5	12.3–13.2	14.3–15.3	11.5–11.7	11.3–12.0	12.5–13.2	11.2–11.5	17.2–17.9	0–0.8								
**10**	***Chiloglanisasperocutis* sp. nov.**	10.6–12.5	11.5–12.5	12.6–13.8	10.1–10.6	11.5–12.0	10.6–11.3	12.4–12.9	16.2–18.5	11.8–13.5	0–1.7							
**11**	*C.* sp. “Pungwe”	11.6–13.3	12.8–13.3	13.3–13.8	11.2–11.5	11.7–12.0	10.6–11.3	12.0–12.4	16.9–17.4	12.5–13.5	1.7–3.3	0–0.2						
**12**	*C.* sp. “Zambezi”	11.1–13.3	11.8–13.0	13.1–13.8	11.0–11.5	11.1–11.5	10.6–11.5	11.9–12.4	16.2–16.9	12.0–13.0	2.7–4.1	2.1–3.1	0–1.3					
**13**	*C.* sp. “Shire”	11.6–12.3	11.6–11.8	11.9–12.3	10.3–10.5	10.4–10.6	10.8–11.3	11.2–11.4	15.7–16.2	12.5–13.2	4.7–5.8	5.4–5.8	5.4–6.2	0–0.2				
**14**	*C.* sp. “Nyangombe”	14.5–15.8	13.7–14.2	15.9–16.6	12.5–12.7	11.8–12.5	12.5–13.2	11.3–11.5	16.4–16.9	4.1–4.8	10.0–11.4	11.1–11.6	10.0–10.9	11.3–11.8	0–0.2			
**15**	*A.savorgnani*	18.2–19.0	17.5–18.3	19.2–19.8	17.3–17.8	17.8–18.1	17.5–18.1	17.2–17.5	17.1–17.8	18.5–19.0	17.5–19.2	18.3–19.2	17.6–18.9	16.5–17.0	18.3–19.4	0–1.2		
**16**	*E.boulengeri*	17.0–18.0	17.5–18.0	16.7–16.9	17.2–17.2	17.0–17.6	17.1–17.6	17.3–17.3	14.9–15.2	17.8–18.0	16.5–18.1	16.8	17.3–18.1	16.5–16.7	17.6–17.8	12.8–13.0	_	
**17**	*E.royauxi*	18.5–18.8	17.8–18.3	17.9–18.1	19	19.3–19.8	19.3–20.1	19.5	15.1–15.6	19.5–20.3	18.0–20.2	18.5	19.1–20.2	17.2–17.4	19.3–19.6	12	7.1	_

### ﻿Morphological analysis

**Figure 5.**
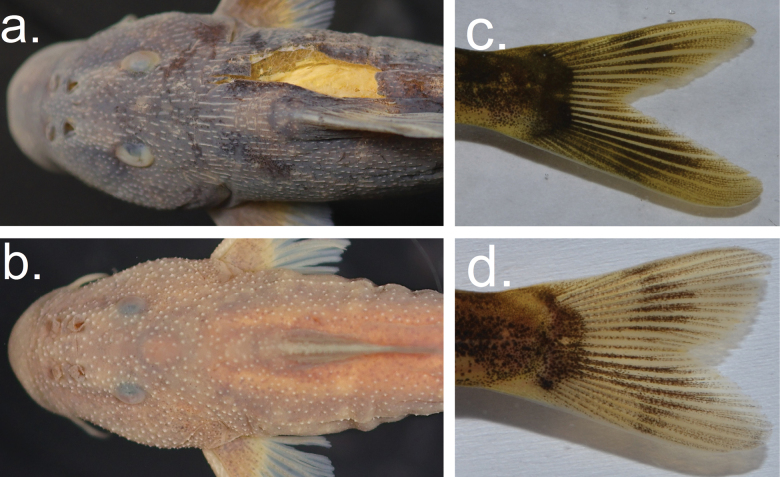
Some of the readily identifiable diagnostic characters separating *Chiloglanisasperocutis* from *C.compactus*. Ridge-like tubercles on the dorsum of **a***C.asperocutis* and conical tubercles on **b***C.compactus*. Deeply forked caudal peduncle with pointed lobes in **c***C.asperocutis* and shallow fork with rounded lobes in **d***C.compactus*.

Detailed morphological examination revealed a number of phenotypic characters that consistently separate *Chiloglanis* sp ‘rough skin’ and *Chiloglanis* sp. ‘dwarf’ from all the eight formally described and allopatrically distributed species from southern Africa. These characters include the caudal peduncle depth, dorsal-fin base length, lower lip length, mandibular tooth row width, upper lip length, number of pectoral-fin rays, number of dorsal-fin rays, number of mandibular teeth, number of primary premaxillary teeth, shape and depth of the caudal fin and the shape of the tubercles (Fig. 5, Table 2).

**Table 2.** Morphological characters used to distinguish *Chiloglaniscompactus* sp. nov. and *Chiloglanisasperocutis* sp. nov. from the valid *Chiloglanis* species found in southern Africa.

Species	*Chiloglaniscompactus* sp. nov.	*Chiloglanisasperocutis* sp. nov.	*Chiloglaniscarnatus*	*Chiloglanispretoriae*	*Chiloglanisanoterus*	*Chiloglanisbifurcus*	*Chiloglanisemarginatus*	*Chiloglanisfasciatus*	*Chiloglanisparatus*	*Chiloglanisswierstrai*
Number of specimens	55	110	19	16	1.0	10	10	10	2	7
Total length	28.9–57.2	32.3–87.6	45.3–62.2	31.7–67.1	80.1	68.7–84.9	50.2–66.6	37.7–53.3	44–51.9	45.2–65.7
Standard length	26.0–45.9	33.9–66.6	35.5–48.9	26.5–54.6	61.7	51.4–63.9	40.3–55.6	30.3–41.7	35.8–42.4	34.9–51.9
Head length	7.1–14.4	11.2–22.8	12.1–15.6	8.8–19.4	20.3	15.7–19.5	12.3–15.7	10.0–13.7	11.1–13.8	9.2–13.6
% Standard length										
Caudal peduncle depth	10.0–12.4	7.5–10.0	11.3–13.2	11.0–13.8	12.2	11.1–14.1	10.2–11.9	7.5–8.8	9.6–9.9	7.2–8.7
Dorsal-fin base length	9.9–14.2	7.9–13.4	10.7–14.1	12.8–18.0	8.6	9.5–13.2	8.8–12.9	10.4–13.7	12.8–14.6	7.5–9.6
% Head length										
Lower lip length	17.5–22.6	18.4–27.4	18.3–26.6	22.4–27.7	25.1	17.7–25.6	23.5–28.8	18.8–24.9	22.8–27.6	19.2–26.0
Mandibular tooth row width	5.6–10.4	3.6–7.2	4.6–8.1	16.0–25.6	10.5	10.4–17.3	9.6–13.5	4.8–6.6	7.2–7.7	10.0–16.6
Upper lip length	6.9–17.1	11.4–22.2	11.1–16.2	11.7–18.8	16.7	8.4–12.3	6.6–10.6	8.8–15.5	12.3–12.4	7.0–10.5
Meristics										
Pectoral-fin rays	7	8	8 (6–8)	8	8.0	8 (7–8)	7	8	8	8
Dorsal-fin rays	5	6 (6–7)	6 (5–7)	6	5.0	5 (5–6)	6 (5–6)	6 (5–6)	5	5 (5–6)
Mandibular teeth	8 (6–8)	10 (8–10)	10	12	12.0	8	8 (6–8)	8	12	11 (11–14)
Primary premaxillary teeth	38 (31–53)	80 (68–128)	60 (43–69)	51–59	86.0	54 (50–64)	36–54	64 (51–65)	39–51	50 (34–59)

### ﻿Taxonomic decisions

Based on the genetic and morphological divergence of *Chiloglanis* sp. ‘rough skin’ and *Chiloglanis* sp. ‘dwarf’ from all the formally described congeners from southern Africa and the other candidate species identified from the Eastern Zimbabwe Highlands freshwater ecoregion, below we provide formal descriptions of these two species as *Chiloglanisasperocutis* sp. nov. and *Chiloglaniscompactus* sp. nov., respectively. The other lineages identified from the previous and present study will be considered for description in a future study once we have gathered additional material for detailed morphological examination.

### ﻿Taxonomic accounts

#### ﻿ Chiloglanis asperocutis

Taxon classificationAnimaliaSiluriformesMochokidae

Mutizwa, Bragança & Chakona sp. nov.

11ECD2A0-E470-5006-952C-460FD7B53CF1

https://zoobank.org/0DDBD844-CD0F-432C-9AD0-E11FE7C7095A

Figs 6 , 7

Chiloglanis sp. “rough skin”: Chakona et al. 2018: 76–79.

##### Type material.

***Holotype*.** • SAIAB 246255, female specimen 66.0 mm SL, Honde River, Bridge on road to Honde Mission, Pungwe River system, Manicaland Province, Zimbabwe, 18.5438°S, 32.8044°E, 13 December 2014, Albert Chakona, Wilbert Kadye and Taurai Bere, genseq-1 COIMH432036. ***Paratypes*.** • SAIAB 201026, 14 male, 29 female specimens (32.3–77.5 mm SL), Honde River, Pungwe River system, Bridge on road to Honde Mission, Manicaland Province, Zimbabwe, 18.5438°S, 32.8044°E, 13 December 2014, Albert Chakona, Wilbert Kadye and Taurai Bere.

##### Other material examined.

Specimens detailed in Suppl. material 1.

##### Diagnosis.

A higher number of primary premaxillary teeth (68–128) readily distinguishes *C.asperocutis* sp. nov. from all its congeners from southern African that consistently have fewer than 68 primary premaxillary teeth except for *C.anoterus* and *C.carnatus*Mutizwa et al., 2024. Possession of ten closely packed mandibular teeth further distinguishes *C.asperocutis* from *C.fasciatus* Pellegrin, 1936 that has eight closely packed mandibular teeth; *C.compactus* sp. nov., *C.bifurcus* and *C.emarginatus* that have eight widely spaced mandibular teeth; *C.anoterus*, *C.paratus* Crass, 1960, and *C.pretoriae* that have 12 closely packed mandibular teeth; and *C.swierstrai* Van der Horst, 1931 that has 14 closely packed mandibular teeth. *Chiloglanisasperocutis* sp. nov. possesses more dorsal fin rays (6–7) compared to *C.compactus* sp. nov. (5 rays). Possession of eight pectoral fin rays distinguishes *C.asperocutis* from *C.compactus* sp. nov. and *C.emarginatus* that possess seven pectoral fin rays. A deeply forked caudal fin readily separates *C.asperocutis* sp. nov. from *C.compactus* sp. nov., *C.pretoriae*, *C.paratus*, and *C.swierstrai* that have shallower fork depths in the caudal fin; *C.emarginatus* that has an emarginated caudal fin with a very shallow fork; and from *C.anoterus* that has a caudal fin with no fork due to extended median rays in males and emarginated in females. A caudal fin with an upper lobe shorter than lower lobe distinguish *C.asperocutis* sp. nov. from *C.bifurcus* that has a caudal fin with an upper lobe that is longer than the lower lobe. An oral disc with a well-developed mid-ventral cleft distinguishes *C.asperocutis* sp. nov. from *C.swierstrai* that lacks a mid-ventral cleft. *Chiloglanisasperocutis* sp. nov. has a dorsal spine with gently serrated anterior and posterior margins that distinguish it from *C.paratus* that has a dorsal spine with a serrated posterior margin. Absence of a fleshy skin on the basal portion of the dorsal fin separates *C.asperocutis* sp. nov. from *C.carnatus*, a character present in the latter species. *Chiloglanisasperocutis* sp. nov. is further distinguished from its southern African congeners by long ridge-like tubercles distributed across the dorsal and lateral surfaces of the head and body compared to conical tubercles found in *C.compactus* sp. nov., *C.bifurcus*, *C.emarginatus*, *C.fasciatus*, *C.paratus*, *C.carnatus*, and *C.swierstrai*; and the lack of conspicuous tubercles in *C.anoterus* and *C.pretoriae*. A narrow caudal peduncle depth distinguishes *C.asperocutis* sp. nov. (7.5–10.0%SL) from *C.carnatus* (11.3–13.2%SL), *C.pretoriae* (11.0–13.8%SL), *C.anoterus* (12.2%SL), *C.bifurcus* (11.1–14.1%SL), and *C.emarginatus* (10.2–11.9%SL) that have deeper caudal peduncles. A narrow mandibular tooth row width separates *C.asperocutis* sp. nov. (3.6–7.2%HL) from *C.pretoriae* (16.0–25.6%HL), *C.anoterus* (10.5%HL), *C.bifurcus* (10.4–17.3%HL), *C.emarginatus* (9.6–13.5%HL), *C.paratus* (7.2–7.7%HL), and *C.swierstrai* (10.0–16.6%HL) that have wider mandibular tooth row widths. A longer upper lip length further separates *C.asperocutis* sp. nov. (11.4–22.2%HL) from *C.emarginatus* (6.6–10.6%HL) and *C.swierstrai* (7.0–10.5%HL) that have shorter upper lips.

##### Description.

**Figure 6.**
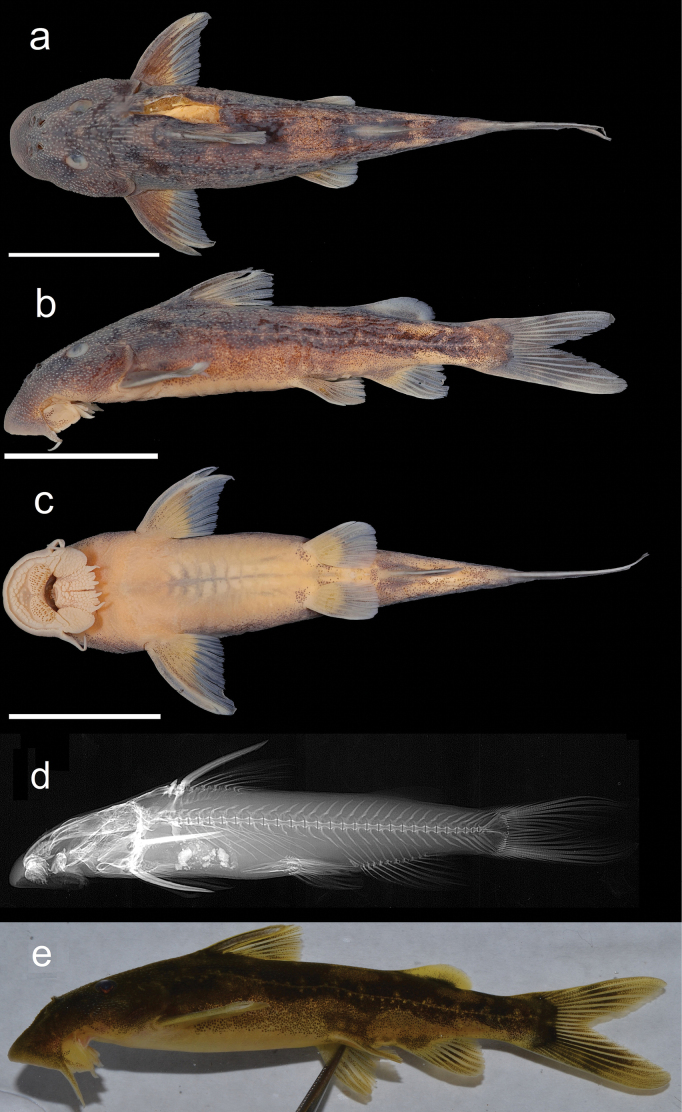
*Chiloglanisasperocutis* female holotype SAIAB 246255. Scale bars: 2 cm.

**Figure 7.**
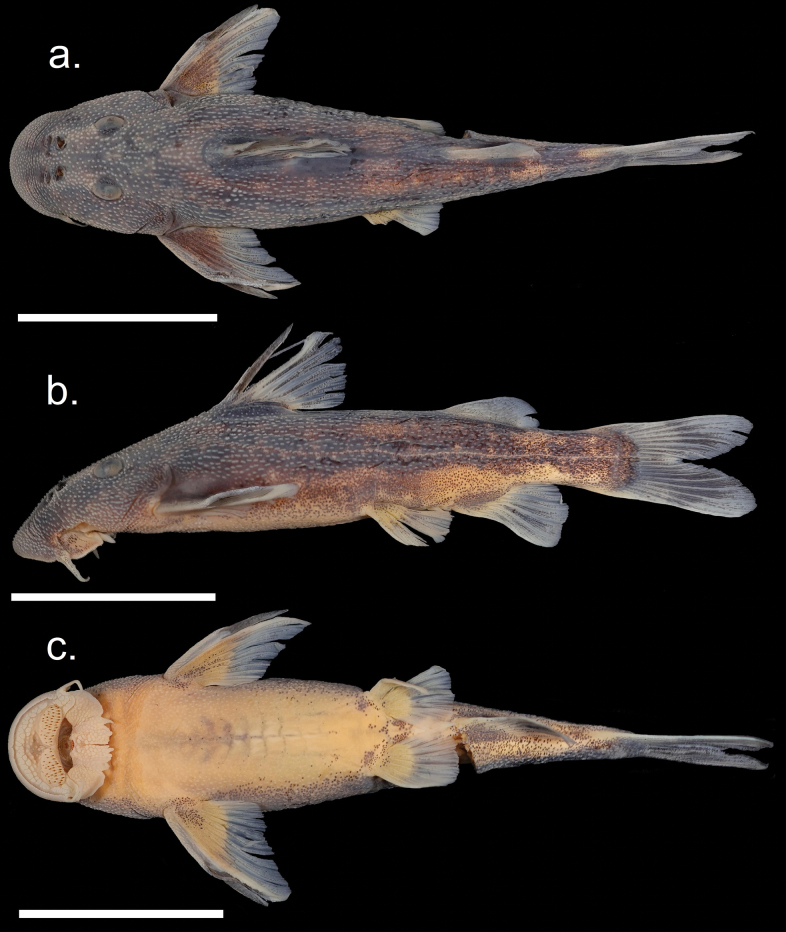
*Chiloglanisasperocutis* male paratype SAIAB 201026. Scale bars: 2 cm.

Figs 6, 7 shows general body features of adult male and female *C.asperocutis*. Morphometric and meristic data for holotype, paratypes, and non-type specimens are presented in Table 3. Where meristics counts have a range, counts from the holotype are given in parentheses.

**Table 3.** Summary of the *Chiloglanisasperocutis* sp. nov. morphological characters. All measured values, except standard length (SL) and Head length (HL) are given as percentages of the HL or SL. The numbers in the parentheses represent the mean of the morphometric characters and the mode for the meristic characters.

	*Chiloglanisasperocutis* holotype	*Chiloglanisasperocutis* paratypes	*Chiloglanisasperocutis* non–types
SAIAB catalogue number	201026	201026	_
Number of specimens	1	42	67
Total length	81.6	32.3–77.5	43.5–87.6 (65.1)
Head length	21.5	17.8–21.5	11.2–22.8 (16.8)
Standard length	66	61.4–66	33.9–66.6 (51.7)
% **standard length**			
Adipose fin to caudal peduncle length	17.6	16–17.6	13.5–21.6 (16.4)
Adipose-fin base length	11.7	11.7–14.5	10.4–17.9 (13.7)
Adipose-fin height	4.7	4.7–4.9	3.9–7 (5.4)
Anal-fin base length	9.7	9.7	8.2–13.2 (10.9)
Anal-fin length along longest ray	12.1	12.1–16.1	10.9–18.9 (14)
Body depth at anus	12.8	12.8–15.4	12.5–16.9 (14.8)
Body depth at dorsal-fin insertion	19.6	18.1–19.6	17.2–22.8 (19.4)
Caudal peduncle depth	8.1	8.1–8.4	7.5–10 (8.9)
Caudal peduncle length	19.1	18.8–19.1	13.9–21.3 (17.4)
Dorsal fin to adipose fin length	26.4	24.9–26.4	18.7–29.2 (24.6)
Dorsal-fin base length	12.4	11.6–12.4	7.9–13.4 (10.2)
Dorsal-spine length	19.4	16.7–19.4	13.8–25.4 (17.5)
Pre-anal length	73.6	70.4–73.6	68.4–77.6 (72.8)
Pre-dorsal length	42.5	39.2–42.5	37.6–46.3 (40.9)
Pre-pectoral length	29	26.5–29	25.7–33 (29.7)
Pre-pelvic length	57.8	57.1–57.8	53.6–61.8 (58.8)
Pectoral-spine length	20.8	20.8–21.3	13.4–26.6 (20.2)
Pectoral-fin length	21.8	21.8–25.5	19.4–27.4 (22.7)
Pelvic-fin length	14.3	13.2–14.3	11.7–17.5 (14.6)
Width at pectoral-fin insertion	23.9	22.7–23.9	21–27.1 (23.9)
Pelvic-fin interspace	4.8	4.8–5.4	2.8–9.2 (5.1)
Head length	32.6	29–32.6	28.6–36.3 (32.6)
Dorsal-fin length along longest ray	20.5	20.5–20.6	12.3–22.8 (17.8)
Caudal fork length	10.3	8.5–10.3	9.4–15.9 (12.5)
% **Head length**			
Anterior nares interspace	13.9	13.4–13.9	10.3–19.5 (14.4)
Eye diameter (vertical axis)	12.5	12.5–13.4	7.9–15.9 (12.7)
Lower lip length	21.3	18.7–21.3	18.4–27.4 (22.4)
Mandibular tooth row width	6.4	6.4–7.2	3.6–6.9 (5.5)
Maxillary barbel length	27.8	23.9–27.8	20.2–38.9 (28.3)
Mouth width	25.9	25.9–31	26.6–34.1 (31)
Orbital interspace	20.8	20.4–20.8	16.5–22.6 (19.9)
Oral disc length	57.2	53.7–57.2	47.1–63.3 (52.6)
Oral disc width	51.7	51.7–59	49.6–69.2 (57.9)
Premaxillary tooth-patch length	10.5	10.1–10.5	7.6–14 (10.6)
Premaxillary tooth-patch width	36.4	36.4–46.8	29.7–51.2 (43.5)
Posterior nares interspace	17.3	10.1–17.3	7.5–14.9 (11.3)
Snout length	65	60.1–65	54.8–68 (63.9)
Upper lip length	13	13–16.8	11.4–22.2 (15.6)
Eye diameter (horizontal axis)	15.7	15.7–17	22.3–51.4 (34.2)
Occipital shield width	36.1	36.1–40.1	48.4–60 (54.2)
Head depth	52	52–55.8	22.5–89.5 (61.6)
**Meristics**			
Pelvic fin count	7	7	7
Pectoral fin count	8	8	8
Primary premaxillary tooth row count	4	3–5	4 (3–5)
Primary premaxillary tooth count total	102	74–102	80 (68–128)
Mandibular tooth count	10	10	10 (8–10)
Dorsal-fin rays	6	6	6 (6–7)
Anal-fin rays	10	10–11	9 (9–11)
Total vertebrate	29	28–29	27 (27–29)
Abdominal vertebrae	13	11–13	12 (11–13)
Caudal vertebrae	16	15–17	15–17

***Body*** elongate. Depressed pre-dorsal region, mid-sections more cylindrical, slightly laterally compressed posterior section. Pre-dorsal profile convex, sharply slopping from snout to posterior nostril, gentler slope from poster nostril to dorsal-fin origin. Body greatest depth at dorsal-fin insertion. Post-dorsal profile almost straight to adipose fin insertion, gently concave from adipose-fin origin to caudal fin. Ventral profile gently convex from region just posterior of oral disc to anal-fin origin, gently concave from anal-fin origin to caudal fin.

***Skin*** numerous tubercles spread across body with exception of ventral surface, forming distinct ridge like structures most conspicuous on head dorsum giving skin a distinct “rough” texture. Lateral line complete, originating just above horizontal level of orbit, below anterior dorsal fin origin, gently undulating along midlateral of body to base of caudal fin.

***Head*** relatively big; longer than body depth; ~ 2/3 of pre-dorsal length. Eye large and oval, orbit lacks free margin; located dorsally, closer to margin of operculum than tip of snout. Interorbital space slightly wider than space between nares spaces. Posterior nares slightly closer together than anterior nares. Anterior nares with short skin flaps on their posterior margins. Posterior nares with short skin flaps along anterior margin. Gill opening restricted, located adjacent to pectoral fin origin.

***Mouth*** inferior, projecting below ventral body surface, upper and lower lips combined into a distinct large oral disc. Oral disc width greater than length. Upper and lower lips with pronounced roundish papillae, largest papillae concentrated around mid-ventral cleft of lower lip. Three unbranched pairs of barbels. Maxillary barbel, originating from lateral region of oral disc, extending to posterior region of oral disc. Lateral mandibular barbel longer than medial mandibular barbel, both integrated into lower lip.

***Dentation*** variable number 68–128 (102) of primary pre-maxillary teeth arranged in 3–5 (4) rows on two ovoid tooth patches. Secondary pre-maxillary teeth small and scattered on posterior surface of premaxillae. Tertiary teeth small and needle-like; in a row near midline of dorsal edge of tooth plate. Up to 10 (10) closely packed mandibular teeth; central teeth projecting higher than outer teeth forming a gentle arc; replacement tooth row emerges anteriorly to functional row, number of mandibular teeth may vary due to loss of teeth and their replacements, but the number does not exceed ten.

***Vertebral counts*** Total vertebrae 27–29 (29), abdominal vertebrae 11–13 (13), caudal vertebrae 15–17 (16).

***Urogenital papillae*** distinctly elongate and sharply pointed in males, reduced to a shallow invagination in females.

***Pectoral fin*** ray count 8, origin adjacent to gill opening; pectoral spine slightly curved, anterior margin smooth; gently serrated posterior margin; pectoral spine length ~80% of longest pectoral-fin ray. ***Dorsal fin*** originating in anterior 1/3 of body, posterior to pectoral-fin origin, 6–7 (6) branched rays. Dorsal spine, length ~ 80% of longest dorsal-fin ray, gentle serrations on anterior and posterior margins of the terminal 1/4 of dorsal spine. ***Pelvic fin*** ray count 7, origin posterior to midpoint between end of dorsal-fin and adipose-fin origin; rounded. ***Adipose fin*** relatively small, margin convex, located in posterior 1/3 of body, origin just anterior of anal-fin origin, ridge-like tubercles sometimes present on its surfaces. ***Anal fin*** ray count 12–13 (13), origin just posterior to origin of adipose fin; terminating just before posterior end of adipose fin; rounded. ***Caudal fin*** deeply forked, lobes unequal, lower lobe generally longer, both lobes with gently pointed tips.

***Coloration in ethanol*** dorsal and lateral skin generally dark brown becoming a lighter shade of brown below mid lateral line. Dark melanophores distributed throughout dorsal and lateral surfaces. Dorsal surface has blotches of lighter shades of brown located between orbits and posterior nostril, at dorsal fin insertion, between dorsal and adipose fin, and posterior to adipose fin, all these blotches extend to lateral surface. Lateral surface with large pale blotches such as those extending from dorsal surface. Lateral line clearly visible as a continuous pale brown stripe with very small pale blotches distributed above and below it. Ventral surface pale cream to yellowish in colour with dark melanophores only present at base of pelvic fins. Dorsal and adipose fins dark brown base and middle with hyaline margins. Pectoral fins have dark brown dorsal surfaces and lighter ventral surface, hyaline margins. Pelvic and anal fins pale brown in colour with dark melanophores and hyaline margins. Dark band stretches across middle of upper caudal fin lobe, lower lobe mostly covered by a dark blotch originating from the fin base. ***Live colouration*** displayed similar patterns as described in persevered specimens with a few differences. All pale brown sections in live specimens have a golden colour. Live specimens display a range of pigmentation intensity, some specimens are very dark with patterns described in the ***Coloration in ethanol*** section being very clear whilst other specimens are lightly coloured (golden) with little pigmentation visible.

***Reproduction***: there are no dedicated studies on the breeding biology of *C.asperocutis*, but spawning is likely to begin in summer (October–November) based on the general pattern of other congeners (Skelton 2001), and other mochokid fishes from this region.

##### Distribution, habitat, and feeding ecology.

this species is known from multiple localities in the Pungwe and Buzi River systems. It is a rheophilic species that occurs in rocky habitats with fast flowing water. Macroinvertebrates from the families Simuliidae, Chironomidae, Hydropsychidae and Libellulidae were the dominant prey item for this species with algae forming a smaller component of the diet of this species (Matomela et al 2018).

##### Etymology.

a combination of the Latin words, *aspero*, meaning rough, and *cutis*, meaning skin. The name refers to the distinct ridges on the skin (more pronounced on the head dorsum) which is characteristic of this species.

#### ﻿ Chiloglanis compactus

Taxon classificationAnimaliaSiluriformesMochokidae

Mutizwa, Bragança & Chakona sp. nov.

63245AD1-94A7-555D-9175-1FCC90425310

https://zoobank.org/3EEC0C8D-4992-4A8C-9226-1BE59BAAB312

Figs 8 , 9

Chiloglanis sp. “dwarf”: Chakona et al. 2018: 76–79.

##### Type material.

***Holotype*.** • SAIAB 246256, male, right middle cut, 44.4 mm SL, Makanga River Bridge on road to Hauna growth point, Pungwe River system, Manicaland Province, Zimbabwe, 18.5438°S, 32.801°E, 16 December 2014, Albert Chakona, Wilbert Kadye and Taurai Bere, genseq-1 COIMH432044. ***Paratypes*.** • SAIAB 210377, 10 males (29.8–37.5 mm SL), 2 females (29.1–40.3 mm SL), Makanga River bridge on road to Hauna growth point, Pungwe River system, Manicaland Province, Zimbabwe, 18.5438°S, 32.801°E, 16 December 2014, Albert Chakona, Wilbert Kadye and Taurai Bere.

##### Other material examined.

Specimens detailed in Suppl. material 1.

##### Diagnosis.

*Chiloglaniscompactus* sp. nov. attains the smallest size (< 46 mm SL) for all congeners currently known from southern Africa. Possession of seven pectoral fin rays in *C.compactus* sp. nov. distinguishes this species from *C.asperocutis*, *C.carnatus*, *C.anoterus*, *C.fasciatus*, *C.paratus*, *C.pretoriae*, and *C.swierstrai* that all have eight pectoral fin rays. Possession of eight widely spaced mandibular teeth further distinguishes *C.compactus* sp. nov. from *C.fasciatus* (eight closely packed mandibular teeth); *C.asperocutis* and *C.carnatus* (ten closely packed mandibular teeth); *C.anoterus*, *C.paratus*, and *C.pretoriae* (12 closely packed mandibular teeth); and *C.swierstrai* (14 closely packed mandibular teeth). Fewer primary premaxillary teeth in *C.compactus* sp. nov. (31–53) readily distinguished it from *C.asperocutis* (68–128) and *C.anoterus* (86). Fewer dorsal fin rays (5) readily distinguish *C.compactus* sp. nov. from *C.asperocutis* (6–7). A relatively shallow forked tail and rounded caudal fin lobes readily separate *C.compactus* sp. nov. from *C.asperocutis*, *C.carnatus*, and *C.fasciatus* (deeply forked caudal fin with pointed lobes); *C.emarginatus* (emarginated caudal fin with very shallow fork); and from *C.anoterus* that has a caudal fin with no fork due to extended median rays in males and emarginated in females. A lower caudal fin lobe longer than the upper lobe separates *C.compactus* sp. nov. from *C.bifurcus* that has a longer upper caudal fin lobe. A well-developed mid-ventral cleft in the oral disc of *C.compactus* sp. nov. distinguishes it from *C.swierstrai* that lacks a mid-ventral cleft. Gently serrate anterior and posterior margins of the dorsal spine in *C.compactus* sp. nov. distinguish it from *C.paratus* that has a dorsal spine serrated only on the posterior margin. Absence of a fleshy skin on the basal portion of the dorsal fin separates *C.compactus* sp. nov. from *C.carnatus*, a character present in the latter species. Conical tubercles distributed across dorsal and lateral surfaces of the head and body distinguish *C.compactus* sp. nov. from *C.asperocutis* with ridge like tubercles; *C.anoterus* and *C.pretoriae* that lack conspicuous tubercles. A deep caudal peduncle distinguishes *C.compactus* sp. nov. (10.0–12.4%SL) from *C.asperocutis* (7.5–10.0%SL), *C.fasciatus* (7.5–8.8%SL), *C.paratus* (9.6–9.9%SL), and *C.swierstrai* (7.2–8.7%SL) that have narrower caudal peduncles. A longer dorsal-fin base separates *C.compactus* sp. nov. (9.9–14.2%SL) from *C.swierstrai* (7.5–9.6%SL) and *C.anoterus* (8.6%SL) that possess shorter dorsal-fin bases. Shorter lower lips distinguish *C.compactus* sp. nov. (17.5–22.6%HL) from *C.pretoriae* (22.4–27.7%HL), *C.anoterus* (25.1%HL), *C.emarginatus* (23.5–28.8%HL), and *C.paratus* (22.8–27.6%HL) that have longer lower lips. A narrow mandibular tooth row separates *C.compactus* sp. nov. (5.6–10.4%HL) from C.*asperocutis* (3.6–7.2%HL), *C.pretoriae* (16.0–25.6%HL), *C.bifurcus* (10.4–17.3%HL), and *C.swierstrai* (10.0–16.6%HL) that possess greater mandibular tooth row widths.

##### Description.

**Figure 8.**
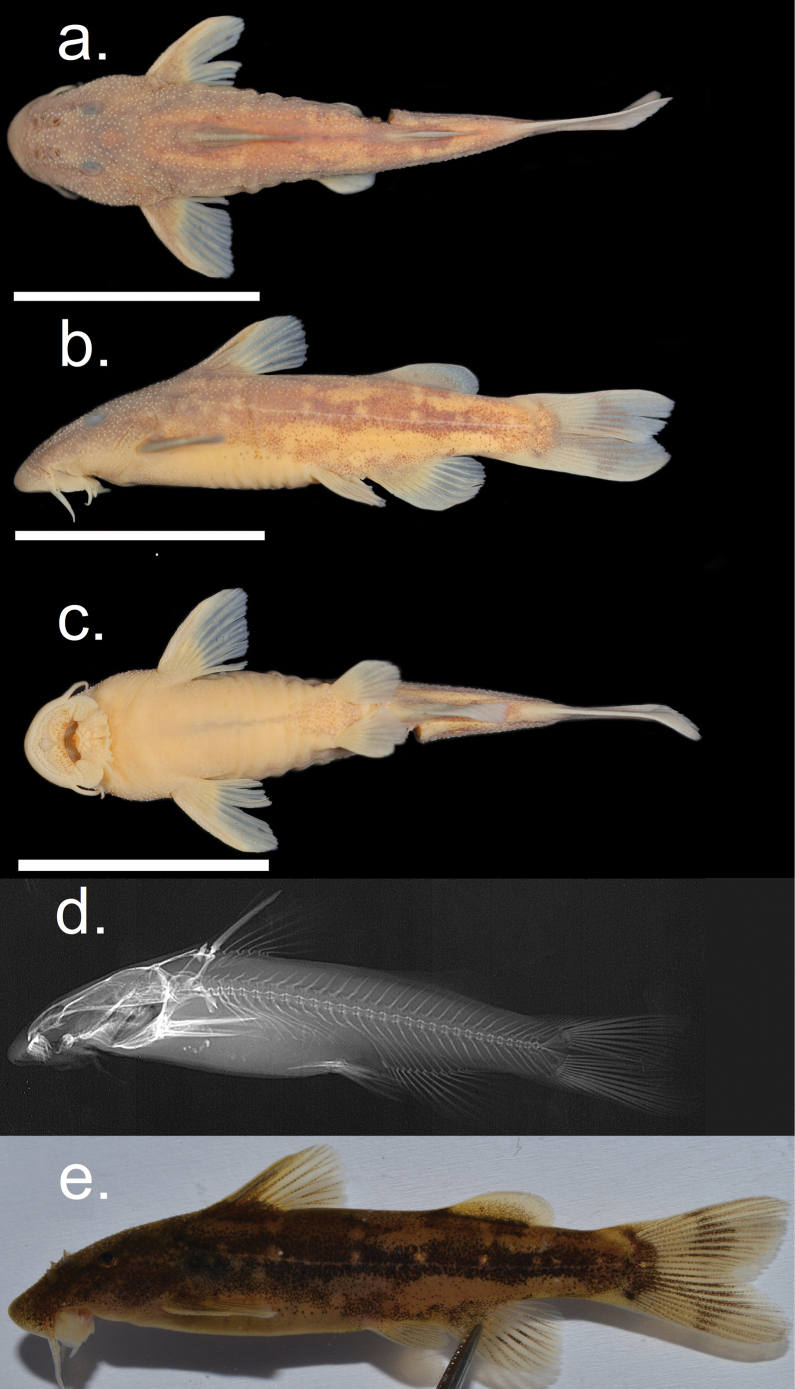
*Chiloglaniscompactus* male holotype SAIAB 246256. Scale bars: 2 cm.

**Figure 9.**
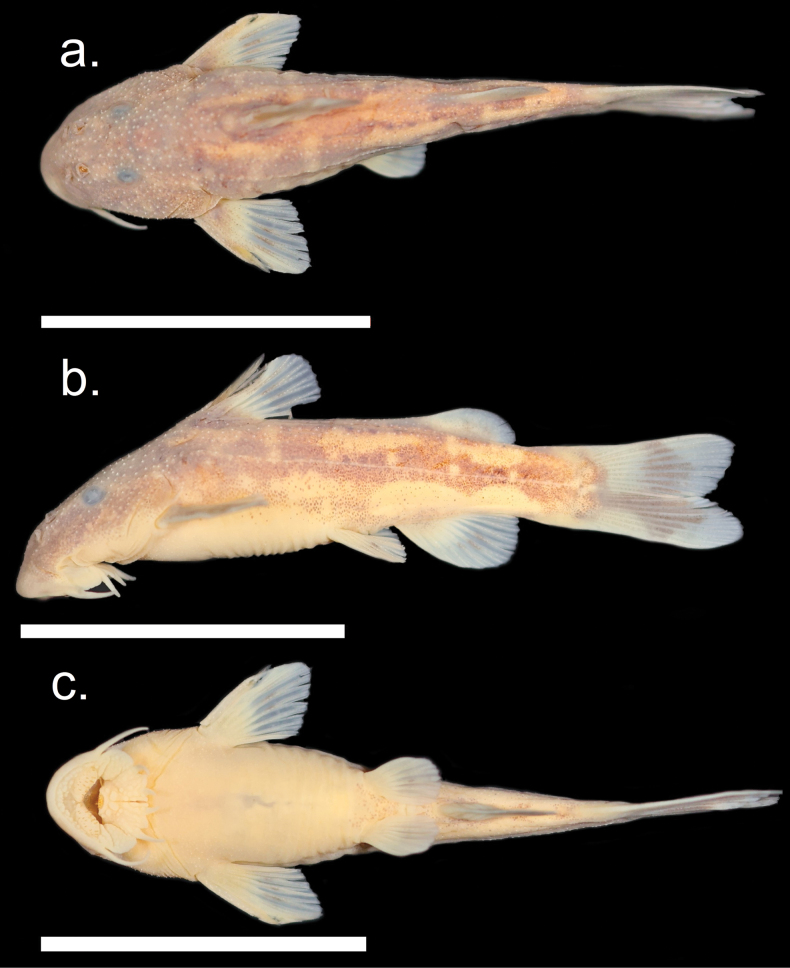
*Chiloglaniscompactus* female paratype SAIAB 210377. Scale bars:2 cm.

Figs 8, 9 shows general body features of adult male and female *C.compactus*. Morphometric and meristic data for holotype, paratypes and non-type specimens are presented in Table 4. Where meristics counts have a range counts from the holotype are given in parentheses.

**Table 4.** Summary of *Chiloglaniscompactus* sp. nov. morphological characters. All measured values, except standard length (SL) and Head length (HL) are given as percentages of the HL or SL. The numbers in the parentheses represent the mean of the morphometric characters and the mode for the meristic characters.

	*Chiloglaniscompactus* holotype	*Chiloglaniscompactus* paratypes	*Chiloglaniscompactus* non–types
SAIAB catalogue number	210377	210377	_
Number of specimens	1	12	42
Total length	42.7	29.8–37.5	31.4–57.2 (44.1)
Head length	11.5	9.1–11.7	7.1–14.4 (11.3)
Standard length	34	28.5–41.2	26–45.9 (35.2)
% **standard length**			
Adipose fin to caudal peduncle length	16.2	16.2–20	11.3–19.8 (16)
Adipose-fin base length	17.3	16.2–18.4	13.2–19.6 (15.7)
Adipose-fin height	4.6	4.6–5.2	2.8–7.1 (5.2)
Anal-fin base length	14.8	10.6–17	10.6–16.4 (13.5)
Anal-fin length along longest ray	18.1	14.9–18.1	11.8–20.5 (16)
Body depth at anus	16.9	16.1–17.2	14.3–20.8 (17.9)
Body depth at dorsal-fin insertion	20.9	20–21.3	16.8–25.3 (21)
Caudal peduncle depth	11.1	10.7–11.1	10–12.4 (11.4)
Caudal peduncle length	18.7	18.7–26.2	15.6–24.5 (19.9)
Dorsal fin to adipose fin length	26	24.6–31.2	19.5–30.2 (24.2)
Dorsal-fin base length	13.5	11.1–14.2	9.9–11.8 (11.1)
Dorsal-spine length	16.3	14.7–19.3	11.6–21.6 (15.7)
Pre-anal length	64.4	64.1–66.5	60.9–71 (67.1)
Pre-dorsal length	39.9	39.8–40.7	35.4–46.2 (39.7)
Pre-pectoral length	29.1	28.8–29.1	24.3–33.6 (29.3)
Pre-pelvic length	55.5	53.8–57.1	51.5–59.3 (55.8)
Pectoral-spine length	18	14.9–18	13.7–21 (17.1)
Pectoral-fin length	23.8	21.7–23.8	15.4–25.6 (21.2)
Pelvic-fin length	13.2	12.7–13.6	10.2–18 (13.7)
Width at pectoral-fin insertion	23.6	23–24.8	21.6–27.6 (24.2)
Pelvic-fin interspace	4.5	4.3–4.5	1.8–7.7 (4)
Head length	33.8	28.3–33.8	25.9–34.9 (31.9)
Dorsal-fin length along longest ray	23.7	17.9–23.7	7.6–22.4 (15.6)
Caudal fork length	15.9	15.4–16.8	12.4–24.5 (17.5)
% **Head length**			
Anterior nares interspace	12.5	12.5–17.3	10.9–23 (16.4)
Eye diameter (vertical axis)	10.9	10.9–13.7	11.7–18 (15)
Lower lip length	18.5	18.5–22.6	17.5–21.3 (19.1)
Mandibular tooth row width	5.6	5.6–6.9	5.9–10.4 (8.6)
Maxillary barbel length	22.2	22.2–37.6	17.4–45.7 (30.6)
Mouth width	25.8	25.8–32.7	28.2–35.1 (31)
Orbital interspace	20.7	20.7–23.6	24.7–27.5 (25.7)
Oral disc length	46.4	46.4–56.5	38.6–54.7 (47.6)
Oral disc width	47.3	47.3–57.4	43.8–69.7 (51.7)
Premaxillary tooth-patch length	6.9	6.9–11	5.4–14.3 (8.6)
Premaxillary tooth-patch width	39.9	39.9–47.9	29.8–46.8 (38.9)
Posterior nares interspace	17.8	17–17.8	12.8–20 (15.6)
Snout length	51.9	51.9–65.3	51.1–69.7 (57.4)
Upper lip length	9.6	9.6–12.1	6.9–17.1 (12.7)
Eye diameter (horizontal axis)	13.5	13.5–16.5	22.9–46 (35.5)
Occipital shield width	37.2	37.2–53.1	68.7–83.6 (74.3)
Head depth	55.6	55.6–60.2	59.2–82.3 (69.7)
**Meristics**			
Pelvic fin count	7	7	7
Pectoral fin count	7	7	7
Primary premaxillary tooth row count	3	2–3	2 (2–3)
Primary premaxillary tooth count total	44	42–47	38 (31–53)
Mandibular tooth count	6	6	8 (6–8)
Dorsal-fin rays	5	5	5
Anal-fin rays	10	9–10	10 (9–12)
Total vertebrate	28	27–29	29 (27–29)
Abdominal vertebrae	11	11–12	11 (11–12)
Caudal vertebrae	17	17–18	17 (17–18)

***Body*** short and rotund. Depressed pre-dorsal region, mid-sections more cylindrical, slightly laterally compressed posterior section. Pre-dorsal profile convex, almost continuous rounded profile from snout to dorsal-fin origin. Body greatest depth at dorsal-fin insertion. Post-dorsal profile convex to adipose fin insertion, becoming gently concave from adipose-fin origin to caudal fin. Ventral profile convex from region just posterior of oral disc to anal-fin origin, becoming gently concave from anal-fin origin to caudal fin.

***Skin*** numerous conical tubercles spread across body with exception of ventral surface. Lateral line complete, originating above horizontal level of orbit, below anterior dorsal fin origin, almost completely straight along midlateral of body to base of caudal fin.

***Head*** relatively big; longer than body depth; ~ 2/3 of pre-dorsal length. Eye large and oval, orbit lacks free margin; located dorsally, closer to margin of operculum than tip of snout. Interorbital space wider than space between nares spaces. Posterior nares slightly closer together than anterior nares. Anterior nares with short skin flaps on posterior margin. Posterior nares with short skin flaps along anterior margin. Gill opening restricted, located adjacent to pectoral fin origin.

***Mouth*** inferior, projecting below ventral body surface, upper and lower lips combined into a distinct large oral disc. Oral disc width greater than length. Upper and lower lips with pronounced roundish papillae, largest papillae concentrated around mid-ventral cleft of lower lip. Three pairs of barbels. Maxillary barbel unbranched, originating from lateral region of oral disc, extending to posterior region of oral disc. Lateral mandibular barbel longer than medial mandibular barbel, both integrated into lower lip.

***Dentation*** variable number 31–53 (44) of primary pre-maxillary teeth arranged in 2–3 (3) rows on two ovoid tooth patches. Secondary pre-maxillary teeth small and scattered on posterior surface of premaxillae. Tertiary teeth small and needle-like; in a row near midline of dorsal edge of tooth plate. Up to 8 (6) spaced out mandibular teeth; replacement tooth row emerges anteriorly to the functional row, number of mandibular teeth may vary due to loss of teeth and their replacement, but the number hardly ever exceeds 8.

***Vertebral counts*** Total vertebrae 27–29 (28), abdominal vertebrae 11–12 (11), caudal vertebrae 17–18 (17)

***Urogenital papillae*** distinctly elongate and sharply pointed in males, reduced to a shallow invagination in females.

***Pectoral fin*** ray count 7, origin adjacent to gill opening; pectoral spine slightly curved, anterior margin smooth; gently serrated posterior margin; pectoral spine length ~ 80% of longest pectoral-fin ray. ***Dorsal fin*** originating in anterior 1/3 of body, posterior to pectoral-fin origin, five branched rays, conical tubercles sometime present on its surfaces. Dorsal spine, length ~ 80% of longest dorsal-fin ray, gentle serrations on anterior and posterior margins of terminal 1/4 of dorsal spine. ***Pelvic fin*** ray count 7, origin posterior to midpoint between end of dorsal-fin and adipose-fin origin; rounded. ***Adipose fin*** relatively small, margin convex, located in posterior 1/3 of body, origin just anterior of anal-fin origin, conical tubercles sometimes present on its surfaces. ***Anal fin*** ray count 12–13 (13), origin just posterior to origin of adipose fin; terminating just before posterior end of adipose fin; rounded. ***Caudal fin*** with shallow fork, lobes unequal with lower slightly longer, both lobes rounded, conical tubercles sometime present on their surfaces.

***Coloration in ethanol*** dorsal and lateral skin generally dark brown becoming a lighter shade of brown below mid lateral line. Dark melanophores distributed throughout dorsal and lateral surfaces. Dorsal surface has large blotches of lighter shades of brown located at base of dorsal fin, between dorsal and adipose fin, and posterior end of adipose fin; these blotches extend to lateral surface. Lateral surface with large pale blotches such as those extending from the dorsal surface. Additional large blotches on lateral surface are found below lateral line above pelvic fin, anal fin and caudal peduncle. Approximately seven small circular blotches just above lateral line, two or three similar blotches found above each of them. Approximately four small circular blotches below the lateral line distributed from below dorsal fin to pelvic fin. Lateral line clearly visible as a continuous pale brown stripe. Ventral surface pale cream to yellowish in colour with dark melanophores only present at base of pelvic fins and caudal peduncle. Dorsal and adipose fins brown base and middle with hyaline margins. Pectoral fins have dark brown dorsal surfaces and lighter ventral surface, hyaline margins. Pelvic and anal fins pale brown in colour with hyaline margins. Dark band stretches across middle of upper caudal fin lobe, lower lobe mostly covered by a dark blotch originating from the fin base. ***Live colouration*** displayed similar patterns as described in the preserved specimens with a few differences. Live specimens display a range of pigmentation intensity, some specimens are very dark with patterns described in the ***Coloration in ethanol*** section being very clear whilst other specimens are lightly coloured with little pigmentation visible.

***Reproduction***: there have been no dedicated studies on the breeding biology of *C.compactus*, but spawning is likely to begin in summer (October–November) based on the general pattern of other congeners (Skelton 2001), and other mochokid fishes from this region.

##### Distribution, habitat, and feeding ecology.

this species occurred at multiple localities in the Pungwe and Buzi river systems with the majority of the collections occurring at high elevation. It is a rheophilic species that occurs in rocky habitats with fast flowing water. Its diet was not examined.

##### Etymology.

the name is drawn from the word compact which is inspired by the short and rotund body shape of this species as well as it being the smallest of all the currently known congeners in southern Africa.

## ﻿Discussion

Consistent with Marshall’s (2011) proposition regarding the taxonomic uncertainties within the genus *Chiloglanis* in central southern Africa, and the subsequent work by Chakona et al. (2018), this study identified two new suckermouth catfishes that were previously lumped together into a single species, *C.neumanni*. The two newly described species, *C.asperocutis* and *C.compactus*, are sympatrically distributed in the Pungwe and Buzi river systems. The description of these new species from the EHZ, together with the recent discovery of *C.carnatus* in the middle Zambezi River (Mutizwa et al. 2024) and the occurrence of several lineages (e.g., *Chiloglanis* sp. “Pungwe”, *Chiloglanis* sp. “Zambezi”, *Chiloglanis* sp. “Nyangombe” and *Chiloglanis* sp. “Shire”) highlights this region as a potentially overlooked biodiversity hotspot of rheophilic taxa. This is consistent with the presence of several recently discovered rheophilic species in other genera such as *Anoplopterusmarshalli*, *A.leopardus*, *Heteromormyrus* sp. “Pungwe”, *Heteromormyrus* sp. “Buzi”, and *Zaireichthysmonomotapa* that are endemic to the Buzi and Pungwe river systems as well as the tributaries of the Lower Zambezi that drain the EZH ecoregion (Eccles et al. 2011; Mazungula and Chakona 2021; Mutizwa et al. 2021).

Preliminary examination of specimens of the undescribed *Chiloglanis* lineages (e.g., *Chiloglanis* sp. “Zambezi”, *Chiloglanis* sp. “Nyangombe”, and *Chiloglanis* sp. “Shire”) show some consistent characters that distinguish them suggesting that they are distinct species yet to be described. In the present study, we refrained from making taxonomic decisions for these lineages because we require additional specimens, particularly mature specimens for a thorough morphological examination. *Chiloglanisasperocutis* morphologically resembles the sympatric *Chiloglanis* sp. “Pungwe” lineage. The most conservative decision would be to include *Chiloglanis* sp. “Pungwe” under *C.asperocutis*, but these two taxa are genetically divergent. Detailed morphological examination of a larger sample size of mature specimens, as well as additional evaluations, including, for example CT scan or cleared and stained samples may identify diagnostic characters that reliably distinguish these two taxa.

The discovery of high levels of previously unrecognised diversity of suckermouth catfishes with largely endemic distribution patterns, for example *C.compactus* and *C.asperocutis* endemic to the Pungwe and Buzi rivers, has created renewed interest in the biogeography and evolutionary history of the freshwater fishes of this region. The genus *Chiloglanis* is inferred to have originated in the Congo Basin in the mid-Eocene around 47.53 Ma (95% HPD: 44.65–50.8 Ma) and its current wide distribution across the African continent indicates a complex biogeographic history (Day et al 2023). Available evidence from ancestral range reconstruction for *Chiloglanis* suggests that the clade comprising taxa from the Zambezi, East African and Congo Basin ichthyofaunal provinces (Roberts 1975) underwent diversification as a result of multiple dispersal and recolonization events among these regions (Day et al. 2023). This complex biogeographic history is reflected in the close phylogenetic relationships of most southern African species such as *C.compactus*, *C.asperocutis*, *C.carnatus*, *C.anoterus*, *C.pretoriae*, *C.bifurcus*, and *C.emarginatus* with species and lineages from the Zambezi River, whilst a few species such as *C.paratus* and *C.swierstrai* have closer ties to species from the Congo Basin (Day et al. 2023). The polyphyletic relationship between the sympatrically distributed *C.compactus* and *C.asperocutis* also suggests complex speciation process within the EZH potentially influenced by dispersal between the Zambezi and the Pungwe and Buzi rivers. These hypotheses need to be tested using comparative biogeographic approaches, particularly integrating phylogenomics to more accurately reconstruct the evolutionary relationships and biogeographic patterns of these species. To achieve this, the resolution of the taxonomic uncertainties and more accurate mapping of species distribution ranges is a fundamental requirement. This study provided evidence of the occurrence of two new species in the EZH, highlighting additional taxonomic inconsistencies and paving the way for future studies.

The new species described in this study are restricted to rivers systems (Pungwe and Buzi rivers) that currently face multiple threats from anthropogenic activities. Rich deposits of precious minerals including diamonds and gold support commercial and artisanal mining activities in the EZH. Alluvial gold extraction, in particular, is carried out mainly along rivers and small streams leading to the destruction of the riparian vegetation, increased sediments loads, altered flow regimes and often mercury contamination (Ndunguru et al. 2006; Clark et al. 2019). Exacerbating the situation is the need for better enforcement of regulations to enable sustainable use of the mineral resources in the EZH (Ndunguru et al. 2006; Spiegel 2015; Clark et al. 2019; Kachena and Spiegel 2019). Additionally, climate change, large scale monoculture plantations and riverbank cultivation often alter physiochemical parameters of river systems negatively impacting aquatic biodiversity in the EZH (Clark et al. 2017, 2019; Mafuwe and Moyo 2020). Furthermore, rainbow trout *Oncorhynchusmykiss* (Walbaum 1792) was introduced into the rivers of the EZH for angling and this species negatively influences both the distribution and abundance of native fish and macro-invertebrate species in the rivers of this region (Kadye et al. 2013). Many of the threats to the environment in the EZH arise due to socio-economic activities, however, there is need to for balancing them with conservation needs in order to ensure long term sustainability of the unique fauna, flora and landscapes that play an important part in supporting the communities in this region.

The EZH is a recognised area of high plant diversity and endemism (van Wyk and Smith 2001; Clark et al. 2017). The present and previous studies have uncovered previously overlooked diversity of endemic rheophilic fishes at a time when critical instream habitats are being degraded by multiple human impacts. This places responsibility on conservation authorities to implement measures to reduce or halt the ongoing impacts on these fragile ecosystems. In addition to its unique biodiversity, this region provides ecosystems services of national importance to both Mozambique and Zimbabwe that include water production, carbon sequestration, tourism, non-forest timber products, commercial forestry and highland agriculture, historical, cultural and spiritual value (Clark et al. 2019). Although the benefits from this region are well establish, to date there has been few studies that rigorously quantify the status of ecosystems in this region and the benefits they provide. To address this, the description of species unique to this region and mapping of their distribution provides a base line for assessing the health of the ecosystems of the EZH. For example, due to their dependence on perennial rheophilic habitats, species from the genus *Chiloglanis* along with other habitat specific species (e.g., aquatic invertebrates) have been used as biological indicators to study habitat integrity and instream flow requirements (Kleynhans 1999; Kadye and Moyo 2008; Rashleigh et al. 2009; Soko and Gyedu-Ababio 2015; Achieng et al. 2021). There is need for raising awareness on the uniqueness of the EZH riverine habitats and their endemic biota with emphasis on promoting transdisciplinary approaches involving representatives from local communities, government, industry, academia, NGOs, and conservationists to identify effective strategies to balance socio-economic development with sustainable management and protection of the unique biodiversity of this previously overlooked freshwater ecoregion.

## Supplementary Material

XML Treatment for Chiloglanis asperocutis

XML Treatment for Chiloglanis compactus

## ﻿Additional information

### Author ORCIDs

Tadiwa I. Mutizwa https://orcid.org/0000-0003-4017-1720

Taurai Bere https://orcid.org/0000-0002-8603-5137

Wilbert T. Kadye https://orcid.org/0000-0002-5273-8360

Pedro H. N. Bragança https://orcid.org/0000-0002-8357-7010

Albert Chakona https://orcid.org/0000-0001-6844-7501

### Data availability

All of the data that support the findings of this study are available in the main text or Supplementary Information.

## Supplementary materials

Supplementary material 1 Additional material of new species Data type docx https://binary.pensoft.net/file/1354372This dataset is made available under the Open Database License (http://opendatacommons.org/licenses/odbl/1.0/). The Open Database License (ODbL) is a license agreement intended to allow users to freely share, modify, and use this Dataset while maintaining this same freedom for others, provided that the original source and author(s) are credited.Tadiwa I. Mutizwa, Taurai Bere, Wilbert T. Kadye, Pedro H. N. Bragança, Albert Chakona
