# Supplementary material for: ﻿Description of two new species of Chiloglanis (Teleostei, Mochokidae) from the Eastern Zimbabwe Highlands freshwater ecoregion: an overlooked hotspot of rheophilic fishes
Source: Zookeys. 2025 Jun 16;1241:261–90. doi: 10.3897/zookeys.1241.138917 (PMC12238958; doi:10.3897/zookeys.1241.138917)
Supplement: Supplementary material 1 — Additional material of new species [file zookeys-1241-261_article-138917__-s001.docx]

**Supplementary file and tables**

**Supplementary file 1. Additional material of new species**

***Chiloglanis asperocutis* Mutizwa, Bragança & Chakona, sp. nov.**

**Other material examined.** SAIAB 200933, 66 specimens (28.0–63.0 mm SL), Nyamukombe River bridge, Sagambe, Manicaland Province, Zimbabwe, 18.3958°S, 32.9707°E, 11 December 2014, Albert Chakona, Wilbert Kadye and Taurai Bere, genseq-3 COI MH432034, MH432058. SAIAB 200955, Zimbabwe, 21 specimens (38.7–63.3 mm SL), Ngarura River, low water bridge on road to Mukwindidza through St Pauls School, Manicaland Province, 18.5474°S, 32.8718°E, 13 December 2014, Albert Chakona, Wilbert Kadye and Taurai Bere, genseq-3 COI MH432052, MH432053, MH432059. SAIAB 201035, 31 specimens (32.1–62.6 mm SL), Rwera River through Marupeti village, Zimbabwe and Mozambique Border, Manicaland Province, Zimbabwe, 18.5434°S, 32.8016°E, 14 December 2014, Albert Chakona, Wilbert Kadye and Taurai Bere, genseq-3 COI MH432037. SAIAB 201047, 15 specimens (38.8–57.4 mm SL), Nyamukombe River Bridge between Chisuko School and Katiyo, Manicaland Province, Zimbabwe, 18.3821°S, 33.0327°E, 14 December 2014, Albert Chakona, Wilbert Kadye and Taurai Bere, genseq-3 COI MH432035. SAIAB 201075, 4 specimens (47.7–66.2 mm SL), Main stream Pungwe River on dirt road off main road between Ruda and Katiyo, Manicaland Province, Zimbabwe, 18.4414°S, 32.8875°E, 16 December 2014, Albert Chakona, Wilbert Kadye and Taurai Bere, genseq-3 COI MH432050. SAIAB 201088, 31 specimens (29.0–57.6 mm SL), Nyamukwarara River next to the Nyamukwarara Clinic, Manicaland Province, Zimbabwe, 18.6918°S, 32.9236 °E, 17 December 2014, Albert Chakona, Wilbert Kadye and Taurai Bere, genseq-3 COI MH432055, MH432056. SAIAB 246256, 3 specimens (37.9–50.2 mm SL), Makanga River Bridge on road to Hauna growth point, Manicaland Province, Zimbabwe, 18.5438°S, 32.8013°E, 16 December 2014, Albert Chakona, Wilbert Kadye and Taurai Bere, genseq-3 COI MH432043. SAIAB 201095, 37 specimens (31.7–61.2 mm SL), Chiyengwa River just before confluence with Nyamukwarara River, Manicaland Province, Zimbabwe, 18.6878°S, 32.922°E, 17 December 2014, Albert Chakona, Wilbert Kadye and Taurai Bere, genseq-3 COI MH432038, MH432039, MH432041, MH432045, MH432051, MH432060. SAIAB 674166, specimens (38.3–45.5 mm SL), Mudzira River below Mahate, Manica Province, Mozambique, 19.6747°S, 33.1906°E, 20 September 2002, Roger Bills, S. Chimela and A. Chivindzi. SAIAB 615885, specimens (22.3–29.6 mm SL), Buzi River, stream on Dombe Road, Manica Province, Mozambique, 19.9356°S, 33.5256°E, 18 August 1999, Roger Bills and P. Yose. SAIAB 85599, 3 specimens (20.5–24.0 mm SL), Bonde River inflow ~ 100m from last riffle, Manica Province, Mozambique, 19.1882°S, 33.0252°E, 15 March 1996, Olaf Weyl. SAIAB 67529, 2 specimens (54.0–55.3 mm SL), ~ 1km upstream from CH5 near camp, Manica Province, Mozambique, 19.6544°S, 33.1933°E, 20 September 2002, Roger Bills, S. Chimela and A. Chivindzi. SAIAB 67545, 15 specimens (26.8–56.3 mm SL), Mussapa Grande River, Manica Province, Mozambique, 19.6311°S, 33.1381°E, 21 September 2002, Roger Bills, S. Chimela and A. Chivindzi. SAIAB 67552, 13 specimens (37.0–67.7 mm SL), Mussapa Pequena River near park “gate”, Manica Province, Mozambique, 19.585°S, 33.0842°E, 22 September 2002, Roger Bills, S. Chimela and A. Chivindzi. SAIAB 67593, 20 specimens (33.2–64.0 mm SL), Mussapa Grande River, Gubvo, Manica Province, Mozambique, 19.6975°S, 33.0411°E, 25 September 2002, Roger Bills, S. Chimela and A. Chivindzi. SAIAB 67603, 17 specimens (36.5–72.0 mm SL), Rotanda River, On main road near Mavita, Manica Province, Mozambique, 19.5431°S, 33.0892°E, 26 September 2002, Roger Bills and S. Chimela. SAIAB 67704, 1 specimen (27.6 mm SL), Mukombe River, Manica Province, Mozambique, 19.9439°S, 33.485°E, 29 September 2002, Roger Bills, S. Chimela and A. Chivindzi. SAIAB 67714, 63 specimens (21.3–45.8 mm SL), Revue River at main road bridge, Manica Province, Mozambique, 19.765°S, 33.9472°E, 20 September 2002, Roger Bills, S. Chimela and A. Chivindzi. SAIAB 85700, 1 specimen (38.8 mm SL), Bonde River causeway above Lake Chicamba, Manica Province, Mozambique, 19.3231°S, 32.9041° E, 12 March 1996, Olaf Weyl. SAIAB 85817, 26 specimens (26.5–42.5 mm SL), Buzi River, Pool below Chicamba Dam wall, Manica Province, Mozambique, 19.1565°S, 33.1454° E, 7 April 1997, Olaf Weyl. SAIAB 85832, 1 specimen (43.0 mm SL), Bonde River causeway above Lake Chicamba, Manica Province, Mozambique,19.3231°S, 32.9041°E, 18 March 1997, Olaf Weyl. SAIAB 85863, 9 specimens (26.0–41.0 mm SL), Bonde River causeway above Lake Chicamba, Manica Province, Mozambique, 19.3231°S, 32.9041°E, 12 July 1996, Olaf Weyl. SAIAB 120763, 2 specimens (36.0–47.0 mm SL), Pungwe River, Gorongosa National Park, Sofala Province, Mozambique, 18.8333°S, 34.5°E, 15 October 1969, M. da Costa. SAIAB 186512, 2 specimens (43.0–54.0 mm SL), Pungwe River, Muanza, Sofala Province, Mozambique, 18.8124°S, 34.7239°E, 4 April 2012, Rob Palmer. SAIAB 236418, 25 specimens (34.0–54.0 mm SL), Pungwe River, Mussambizi Falls, Sofala Province, Mozambique, 19.019°S, 34.4055°E, 25 July 2022, Albert Chakona. SAIAB 236430, 15 specimens (34.0–54.0 mm SL), Pungwe River, Vunduzi River, Sofala Province, Mozambique, 18.6046°S, 34.3351°E, 3 August 2022, Albert Chakona.

***Chiloglanis compactus* Mutizwa, Bragança & Chakona, sp. nov.**

**Other material examined.** SAIAB 200940, 3 specimens (26.0–40.8 mm SL), Pungwe River at Muparutsa next to hydrometric station, Manicaland Province, Zimbabwe, 18.4333°S, 32.8968°E, 12 December 2014, Albert Chakona, Wilbert Kadye and Taurai Bere, genseq-3 COI MH432046, MH432057. SAIAB 200923, 1 specimen (30.0 mm SL), Pungwe River on road to Katiyo, Manicaland Province, Zimbabwe, 18.3955°S, 32.9707°E, 11 December 2014, Albert Chakona, Wilbert Kadye and Taurai Bere, genseq-3 COI MH432061. SAIAB 200952, 2 specimens (33.4–35.9 mm SL), Honde River Bridge, Chikuku, Manicaland Province, Zimbabwe, 18.4337°S, 32.8968°E, 13 December 2014, Albert Chakona, Wilbert Kadye and Taurai Bere, genseq-3 COI MH432047. SAIAB 246356, 5 specimens (35.5–68.3 mm SL), Honde River bridge at St Peters-Jombe School, Manicaland Province, Zimbabwe, 18.5992°S, 32.729°E, 15 December 2014, Albert Chakona, Wilbert Kadye and Taurai Bere, genseq-3 COI MH432054. SAIAB 201095, 39 specimens (32.6–62.0 mm SL), Chiyengwa River just before its confluence with the Nyamukwarara River, Manicaland Province, Zimbabwe, 18.6878°S, 32.922°E, 17 December 2014, Albert Chakona, Wilbert Kadye and Taurai Bere, genseq-3 COI MH432042, MH432048. SAIAB 205074, 3 specimens (25.0–32.0 mm SL), Nyamhingura River, Manicaland Province, Zimbabwe, 18.3696°S, 32.9354°E, 13 December 2013, Albert Chakona, Taurai Bere and Michael Machingura, genseq-3 COI MH432025, MH432026, MH432027. SAIAB 205087, 15 specimens (26.0–43.0 mm SL), Mtarazi River, Bridge on road to Gatsi, below falls, Manicaland Province, Zimbabwe, 18.5324°S, 32.8075°E, 13 December 2013, Albert Chakona, Taurai Bere and Michael Machingura, genseq-3 COI MH432018, MH432019, MH432032. SAIAB 246358, 9 specimens (28.0–50.0 mm SL), Pungwe River to Kayito, Manicaland Province, Zimbabwe, 18.3954°S, 32.9707°E, 13 December 2013, Albert Chakona, Taurai Bere and Michael Machingura, genseq-3 COI MH432030, MH432031. SAIAB 234720, 42 specimens (32.3–77.5 mm SL), Honde River bridge on road to Honde Mission, Manicaland Province, Zimbabwe, 18.5438°S, 32.8044°E, 13 December 2014, Albert Chakona, Wilbert Kadye and Taurai Bere. SAIAB 60022, 18 specimens (17.5–45.8 mm SL), Buzi River, Revue Catchment, Manica Province, Mozambique, 19.3283°S, 32.9294°E, 25 March 1997, Olaf Weyl. SAIAB 246353, 5 specimens (22.3–29.6 mm SL), Buzi River, Stream on Dombe Road, Manica Province, Mozambique, 19.9356°S, 33.5256°E, 18 August 1999, Roger Bills and P. Yose. SAIAB 61897, 1 specimen (35.3 mm SL), Bonde River at causeway, Manica Province, Mozambique, 19.3225°S, 32.9056°E, 30 January 1996, Olaf Weyl. SAIAB 85584, 15 specimens (23.0–30.0 mm SL), Bonde River causeway above Lake Chicamba, Manica Province, Mozambique, 19.3231°S, 32.9042°E, 6 April 1996, Olaf Weyl. SAIAB 85738, 1 specimen (24.0 mm SL), Bonde River causeway above Lake Chicamba, Manica Province, Mozambique, 19.3231°S, 32.9042°E, 4 April 1996, Olaf Weyl. SAIAB 246355, 3 specimens (20.5–24.0 mm SL), Bonde River inflow ca 100 m from last riffle, Manica Province, Mozambique, 19.1882°S, 33.0252°E, 15 March 1996, Olaf Weyl. SAIAB 85660, 3 specimens (21.0–23.0 mm SL), Msika River at bridge, Manica Province, Mozambique, 18.9872°S, 33.0491°E, 5 June 1996, Olaf Weyl. SAIAB 85797, 81 specimens (19.0–28.0 mm SL), Nyamanguena River at bridge, Manica Province, Mozambique, 19.2925°S, 33.0467°E, 5 June 1996, Olaf Weyl. SAIAB 85819, 4 specimens (21.0–29.0 mm SL), Mupandeia River at bridge, Manica Province, Mozambique, 19.1667°S, 32.9667°E, 18 July 1996, Olaf Weyl, SAIAB 85859,1 specimen (25.0 mm SL), Bonde River causeway above Lake Chicamba, Manica Province, Mozambique, 19.3231°S, 32.9042°E, 11 June 1996, Olaf Weyl.

**Tables**

**Table S1.** Collection sites details and voucher catalogue numbers of the specimens included in the genetic analysis. Asterisks (*) denote holotypes.

| **Species name** | **River system** | **Latitude** | **Longitude** | **SAIAB specimen catalogue no.** | **COI sequence ID** |
| --- | --- | --- | --- | --- | --- |
| *Atopochilus savorgnani* | Congo | _ | _ | _ | MK073983 |
| *Atopochilus savorgnani* | Congo | _ | _ | _ | MK073984 |
| *Chiloglanis anoterus* | Nkomati | -25.7567 | 31.4386 | 66158 | LN610269 |
| *Chiloglanis anoterus* | Incomati | -25.7692 | 31.3367 | 69611 | LN610270 |
| *Chiloglanis anoterus* | Incomati | -25.7692 | 31.3367 | 69611 | LN610271 |
| *Chiloglanis anoterus* | Nkomati | -25.8672 | 31.3347 | 69896 | LN610272 |
| *Chiloglanis apserocutis* sp. nov. | Pungwe | -19.019 | 34.4055 | 236418 | PQ424602 |
| *Chiloglanis apserocutis* sp. nov. | Pungwe | -19.019 | 34.4055 | 236418 | PQ424603 |
| *Chiloglanis apserocutis* sp. nov. | Pungwe | -19.019 | 34.4055 | 236418 | PQ424604 |
| *Chiloglanis apserocutis* sp. nov. | Pungwe | -19.019 | 34.4055 | 236418 | PQ424605 |
| *Chiloglanis apserocutis* sp. nov. | Pungwe | -19.019 | 34.4055 | 236418 | PQ424606 |
| *Chiloglanis apserocutis* sp. nov. | Pungwe | -18.6046 | 34.3351 | 236430 | PQ424607 |
| *Chiloglanis apserocutis* sp. nov. | Pungwe | -18.6046 | 34.3351 | 236430 | PQ424608 |
| *Chiloglanis apserocutis* sp. nov. | Pungwe | -18.6046 | 34.3351 | 236430 | PQ424609 |
| *Chiloglanis apserocutis* sp. nov. | Buzi | -19.765 | 33.9472 | 67714 | OQ308653 |
| *Chiloglanis apserocutis* sp. nov. | Pungwe | -18.3958 | 32.9707 | 200933 | MH432034 |
| *Chiloglanis apserocutis* sp. nov. | Pungwe | -18.3958 | 32.9707 | 200933 | MH432058 |
| *Chiloglanis apserocutis* sp. nov. | Pungwe | -18.5474 | 32.8718 | 200955 | MH432052 |
| *Chiloglanis apserocutis* sp. nov. | Pungwe | -18.5474 | 32.8718 | 200955 | MH432053 |
| *Chiloglanis apserocutis* sp. nov. | Pungwe | -18.5474 | 32.8718 | 200955 | MH432059 |
| *Chiloglanis apserocutis* sp. nov.* | Pungwe | -18.5438 | 32.8044 | 246255 | MH432036 |
| *Chiloglanis apserocutis* sp. nov. | Pungwe | -18.5434 | 32.8044 | 201035 | MH432037 |
| *Chiloglanis apserocutis* sp. nov. | Pungwe | -18.3821 | 33.0327 | 201047 | MH432035 |
| *Chiloglanis apserocutis* sp. nov. | Pungwe | -18.4414 | 32.8875 | 201075 | MH432050 |
| *Chiloglanis apserocutis* sp. nov. | Pungwe | -18.6918 | 32.9236 | 201088 | MH432055 |
| *Chiloglanis apserocutis* sp. nov. | Pungwe | -18.6918 | 32.9236 | 201088 | MH432056 |
| *Chiloglanis apserocutis* sp. nov. | Pungwe | -18.6878 | 32.922 | 201095 | MH432038 |
| *Chiloglanis apserocutis* sp. nov. | Pungwe | -18.6878 | 32.922 | 201095 | MH432039 |
| *Chiloglanis apserocutis* sp. nov. | Pungwe | -18.6878 | 32.922 | 201095 | MH432041 |
| *Chiloglanis apserocutis* sp. nov. | Pungwe | -18.6878 | 32.922 | 201095 | MH432045 |
| *Chiloglanis apserocutis* sp. nov. | Pungwe | -18.6878 | 32.922 | 201095 | MH432051 |
| *Chiloglanis apserocutis* sp. nov. | Pungwe | -18.6878 | 32.922 | 201095 | MH432060 |
| *Chiloglanis apserocutis* sp. nov. | Pungwe | -18.5438 | 32.8013 | 210377 | MH432043 |
| *Chiloglanis bifurcus* | Nkomati | -25.815 | 31.2911 | 66375 | OQ308615 |
| *Chiloglanis bifurcus* | Nkomati | -25.815 | 31.2911 | 66375 | OQ308616 |
| *Chiloglanis bifurcus* | Incomati | -25.4303 | 30.7577 | 194837 | MH432062 |
| *Chiloglanis bifurcus* | Incomati | -25.4303 | 30.7577 | 194837 | OL311816 |
| *Chiloglanis carnatus* | Zambezi | -17.4249 | 30.5854 | 211346 | PP156891 |
| *Chiloglanis carnatus* | Zambezi | -17.4249 | 30.5854 | 211346 | PP156892 |
| *Chiloglanis carnatus* | Zambezi | -17.4249 | 30.5854 | 211346 | PP156893 |
| *Chiloglanis carnatus* | Zambezi | -17.4249 | 30.5854 | 211346 | PP156894 |
| *Chiloglanis carnatus* | Zambezi | -17.4249 | 30.5854 | 211346 | PP156895 |
| *Chiloglanis carnatus* | Zambezi | -17.4249 | 30.5854 | 236631 | PP156890 |
| *Chiloglanis compactus* sp. nov. | Pungwe | -18.3955 | 32.9707 | 200923 | MH432061 |
| *Chiloglanis compactus* sp. nov. | Pungwe | -18.4333 | 32.8968 | 200940 | MH432046 |
| *Chiloglanis compactus* sp. nov. | Pungwe | -18.4333 | 32.8968 | 200940 | MH432057 |
| *Chiloglanis compactus* sp. nov. | Pungwe | -18.4337 | 32.8969 | 200952 | MH432047 |
| *Chiloglanis compactus* sp. nov. | Pungwe | -18.5992 | 32.729 | 246356 | MH432054 |
| *Chiloglanis compactus* sp. nov. | Pungwe | -18.6878 | 32.922 | 201095 | MH432042 |
| *Chiloglanis compactus* sp. nov. | Pungwe | -18.6878 | 32.922 | 201095 | MH432048 |
| *Chiloglanis compactus* sp. nov. | Pungwe | -18.3696 | 32.9354 | 205074 | MH432025 |
| *Chiloglanis compactus* sp. nov. | Pungwe | -18.3696 | 32.9354 | 205074 | MH432026 |
| *Chiloglanis compactus* sp. nov. | Pungwe | -18.3696 | 32.9354 | 205074 | MH432027 |
| *Chiloglanis compactus* sp. nov. | Pungwe | -18.3955 | 32.9707 | 246358 | MH432030 |
| *Chiloglanis compactus* sp. nov. | Pungwe | -18.3955 | 32.9707 | 246358 | MH432031 |
| *Chiloglanis compactus* sp. nov. | Pungwe | -18.5324 | 32.8075 | 205087 | MH432018 |
| *Chiloglanis compactus* sp. nov. | Pungwe | -18.5324 | 32.8075 | 205087 | MH432019 |
| *Chiloglanis compactus* sp. nov. | Pungwe | -18.5324 | 32.8075 | 205087 | MH432032 |
| *Chiloglanis compactus* sp. nov.* | Pungwe* | -18.5438 | 32.8013 | 246256 | MH432044 |
| *Chiloglanis emarginatus* | Mbuluzi | -26.1378 | 32.0103 | 66319 | OQ308632 |
| *Chiloglanis emarginatus* | Mbuluzi | -26.1378 | 32.0103 | 66319 | OQ308633 |
| *Chiloglanis emarginatus* | Mbuluzi | -26.1294 | 31.9583 | 67284 | OQ308634 |
| *Chiloglanis emarginatus* | Mbuluzi | -26.1294 | 31.9583 | 67284 | OQ308636 |
| *Chiloglanis emarginatus* | Phongolo | -27.4647 | 31.2789 | 70805 | OQ308635 |
| *Chiloglanis fasciatus* | Okavango | -13.5943 | 16.8805 | 186709 | ANGFW077-12 |
| *Chiloglanis fasciatus* | Okavango | -12.6713 | 16.1114 | 186786 | ANGFW131-12 |
| *Chiloglanis fasciatus* | Okavango | -12.6713 | 16.1114 | 186786 | ANGFW133-12 |
| *Chiloglanis paratus* | Phongolo | -27.5039 | 31.1997 | 70798 | SB8459 |
| *Chiloglanis paratus* | Limpopo | -25.0159 | 31.1191 | 194042 | MPUMA025 |
| *Chiloglanis pretoriae* | Incomati | -26.0281 | 31.1631 | 69631 | OQ308613 |
| *Chiloglanis pretoriae* | Incomati | -26.0281 | 31.1631 | 69631 | OQ308614 |
| *Chiloglanis pretoriae* | Nkomati | -25.9583 | 30.7611 | 70773 | OQ308612 |
| *Chiloglanis pretoriae* | Limpopo | -25.6428 | 26.4136 | 78433 | OL311818 |
| *Chiloglanis pretoriae* | Limpopo | -23.9904 | 31.8258 | 98236 | LN610341 |
| *Chiloglanis pretoriae* | Limpopo | -24.0271 | 30.181 | 203319 | OL311817 |
| *Chiloglanis* sp. "Nyangombe" | Zambezi | -18.2653 | 32.5903 | 210408 | MH432020 |
| *Chiloglanis* sp. "Nyangombe" | Zambezi | -18.2653 | 32.5903 | 210408 | MH432021 |
| *Chiloglanis* sp. "Nyangombe" | Zambezi | -18.2653 | 32.5903 | 210408 | MH432022 |
| *Chiloglanis* sp. "Nyangombe" | Zambezi | -18.2653 | 32.5903 | 210408 | MH432033 |
| *Chiloglanis* sp. "Pungwe" | Pungwe | -18.5992 | 32.729 | 201067 | MH432049 |
| *Chiloglanis* sp. "Pungwe" | Pungwe | -18.6878 | 32.922 | 201095 | MH432040 |
| *Chiloglanis* sp. "Pungwe" | Pungwe | -18.3955 | 32.9707 | 205080 | MH432028 |
| *Chiloglanis* sp. "Pungwe" | Pungwe | -18.3955 | 32.9707 | 205080 | MH432029 |
| *Chiloglanis* sp. "Shire" | Zambezi | -15.0612 | 35.2193 | 118785 | OQ308687 |
| *Chiloglanis* sp. "Shire" | Zambezi | -15.061 | 35.219 | 118785 | MAFW119 |
| *Chiloglanis* sp. "Zambezi" | Okavango | -14.6497 | 16.9066 | 186952 | ANGFW211 |
| *Chiloglanis* sp. "Zambezi" | Zambezi | -17.4863 | 24.2628 | 83857 | OQ308651 |
| *Chiloglanis* sp. "Zambezi" | Zambezi | -12.1755 | 25.2152 | 98163 | OQ308652 |
| *Chiloglanis* sp. "Zambezi" | Okavango | -14.9397 | 17.7188 | 186643 | ANGFW015 |
| *Chiloglanis* sp. "Zambezi" | Okavango | -13.5943 | 16.8805 | 186709 | ANGFW078 |
| *Chiloglanis* sp. "Zambezi" | Nyangombe | -18.0829 | 32.5819 | 200517 | MH432023 |
| *Chiloglanis* sp. "Zambezi" | Nyangombe | -18.0829 | 32.5819 | 200517 | MH432024 |
| *Chiloglanis swierstrai* | Phongolo | -27.4178 | 31.5119 | 70601 | OQ308695 |
| *Chiloglanis swierstrai* | Phongolo | -27.4178 | 31.5119 | 70601 | OQ308693 |
| *Chiloglanis swierstrai* | Phongolo | -27.4178 | 31.5119 | 70601 | OQ308696 |
| *Chiloglanis swierstrai* | Phongolo | -27.4178 | 31.5119 | 70601 | OQ308694 |
| *Euchilichthys boulengeri* | Dipumu | -6.0045 | 22.3905 | _ | HM418085 |
| *Euchilichthys royauxi* | Epulu | _ | _ | _ | KT192823 |

**Table S2.** Morphological characters examined in the present study.

| **Abbreviation** | **Morphological character** |
| --- | --- |
| **AD-CPL** | Adipose fin to caudal peduncle length |
| **ADFBL** | Adipose-fin base length |
| **ADFH** | Adipose-fin height |
| **ANFBL** | Anal-fin base length |
| **ANFL** | Anal-fin length along longest ray |
| **ANI** | Anterior nares interspace |
| **BDA** | Body depth at anus |
| **BDDF** | Body depth at dorsal-fin insertion |
| **CFKL** | Caudal fork length |
| **CPD** | Caudal peduncle depth |
| **CPL** | Caudal peduncle length |
| **DF-ADFL** | Dorsal fin to adipose fin length |
| **DFBL** | Dorsal-fin base length |
| **DFL** | Dorsal-fin length along longest ray |
| **DSL** | Dorsal-spine length |
| **EDH** | Eye diameter horizontal axis |
| **EDV** | Eye diameter vertical axis |
| **HD** | Head depth |
| **HL** | Head length to opercular membrane margin |
| **LLL** | Lower lip length |
| **MTRW** | Mandibular tooth row width |
| **MXBL** | Maxillary barbel length |
| **MW** | Mouth width |
| **OBI** | Orbital interspace |
| **ODL** | Oral disc length |
| **ODW** | Oral disc width |
| **OSW** | Occipital shield width |
| **PANL** | Pre-anal length |
| **PDL** | Pre-dorsal length |
| **PMXL** | Pre-maxillary tooth-patch length |
| **PMXW** | Pre-maxillary tooth patch width |
| **PNI** | Posterior nares interspace |
| **PPTL** | Pre-pectoral length |
| **PPVL** | Pre-pelvic length |
| **PSL** | Pectoral-spine length |
| **PFL** | Pectoral-fin length |
| **PVFL** | Pelvic-fin length |
| **PVI** | Pelvic-fin interspace |
| **SL** | Standard length |
| **SNL** | Snout length |
| **TL** | Total length |
| **ULL** | Upper lip length |
| **WPTFI** | Width at pectoral-fin insertion |
